# Pathogenic Role of mTORC1 and mTORC2 in Pulmonary Hypertension

**DOI:** 10.1016/j.jacbts.2018.08.009

**Published:** 2018-12-31

**Authors:** Haiyang Tang, Kang Wu, Jian Wang, Sujana Vinjamuri, Yali Gu, Shanshan Song, Ziyi Wang, Qian Zhang, Angela Balistrieri, Ramon J. Ayon, Franz Rischard, Rebecca Vanderpool, Jiwang Chen, Guofei Zhou, Ankit A. Desai, Stephen M. Black, Joe G.N. Garcia, Jason X.-J. Yuan, Ayako Makino

**Affiliations:** a Division of Translational and Regenerative Medicine, The University of Arizona College of Medicine, Tucson, Arizona; b State Key Laboratory of Respiratory Disease, Guangzhou Institute of Respiratory Disease, First Affiliated Hospital of Guangzhou Medical University, Guangzhou, China; c Department of Physiology, The University of Arizona College of Medicine, Tucson, Arizona; d Division of Pulmonary, Allergy, Critical Care and Sleep Medicine, The University of Arizona College of Medicine, Tucson, Arizona; e Department of Pediatrics, University of Illinois College of Medicine, Chicago, Illinois; f Division of Cardiology, Department of Medicine, The University of Arizona College of Medicine, Tucson, Arizona

**Keywords:** mTOR, pulmonary hypertension, Raptor, Rictor, right ventricle, EC, endothelial cell, FOXO3a, Forkhead box O3a, GPCR, G protein-coupled receptor, HPH, hypoxia-induced pulmonary hypertension, mTORC1, mammalian target of rapamycin complex 1, mTORC2, mammalian target of rapamycin complex 2, PA, pulmonary artery, PAEC, pulmonary arterial endothelial cell, PAH, pulmonary arterial hypertension, pAKT, phosphorylated AKT, PASMC, pulmonary arterial smooth muscle cell, PDGF, platelet-derived growth factor, PDGFR, platelet-derived growth factor receptor, PH, pulmonary hypertension, PI3K, phosphoinositide 3-kinase, PTEN, phosphatase and tensin homolog, PVR, pulmonary vascular resistance, Raptor, regulatory associated protein of mammalian target of rapamycin, Rictor, rapamycin insensitive companion of mammalian target of rapamycin, RVH, right ventricular hypertrophy, RVSP, right ventricular systolic pressure, SM, smooth muscle, TKR, tyrosine kinase receptor, WT, wild-type

## Abstract

Concentric lung vascular wall thickening due to enhanced proliferation of pulmonary arterial smooth muscle cells is an important pathological cause for the elevated pulmonary vascular resistance reported in patients with pulmonary arterial hypertension. We identified a differential role of mammalian target of rapamycin (mTOR) complex 1 and complex 2, two functionally distinct mTOR complexes, in the development of pulmonary hypertension (PH). Inhibition of mTOR complex 1 attenuated the development of PH; however, inhibition of mTOR complex 2 caused spontaneous PH, potentially due to up-regulation of platelet-derived growth factor receptors in pulmonary arterial smooth muscle cells, and compromised the therapeutic effect of the mTOR inhibitors on PH. In addition, we describe a promising therapeutic strategy using combination treatment with the mTOR inhibitors and the platelet-derived growth factor receptor inhibitors on PH and right ventricular hypertrophy. The data from this study provide an important mechanism-based perspective for developing novel therapies for patients with pulmonary arterial hypertension and right heart failure.

## Highlights

• G protein-coupled receptors and tyrosine kinase receptors signal through the phosphoinositide 3-kinase/Akt/mTOR pathway to induce cell proliferation, survival, and growth. mTOR is a kinase present in 2 functionally distinct complexes, mTORC1 and mTORC2.
• Functional disruption of mTORC1 by knockout of Raptor (regulatory associated protein of mammalian target of rapamycin) in smooth muscle cells ameliorated the development of experimental PH.
• Functional disruption of mTORC2 by knockout of Rictor (rapamycin insensitive companion of mammalian target of rapamycin) caused spontaneous PH by up-regulating platelet-derived growth factor receptors.
• Use of mTOR inhibitors (e.g., rapamycin) to treat PH should be accompanied by inhibitors of platelet-derived growth factor receptors (e.g., imatinib).

## Visual Abstract

**Figure:**
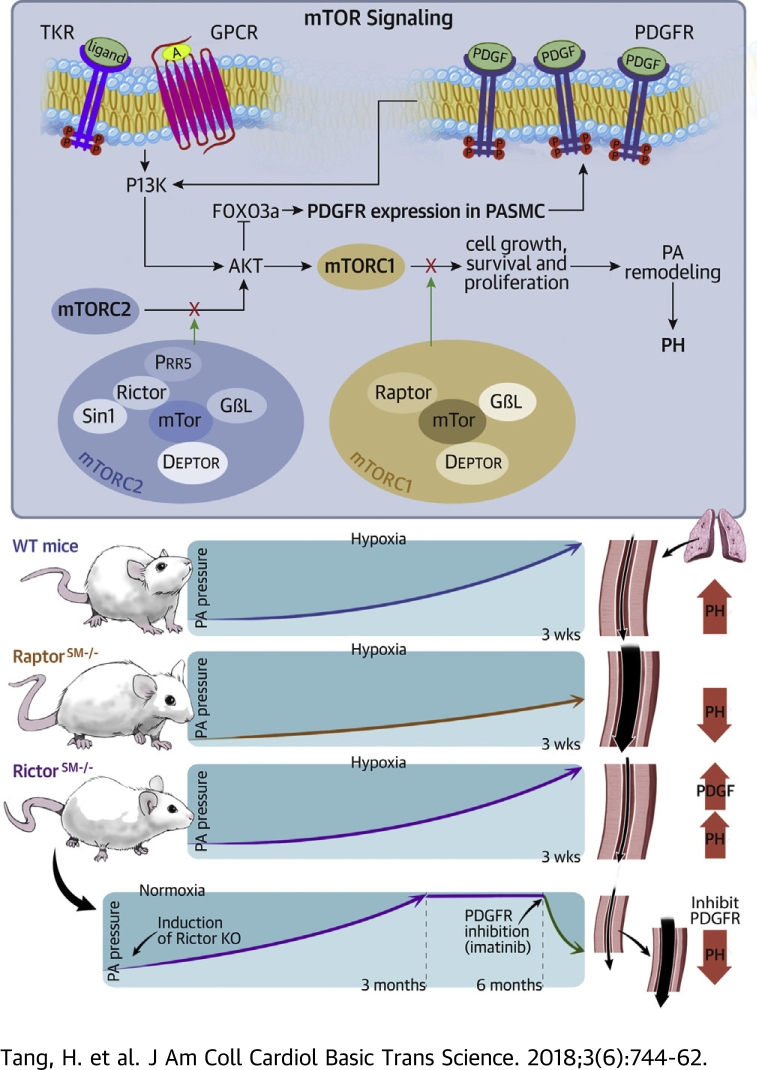

Idiopathic pulmonary arterial hypertension (PAH) is a progressive and fatal disease in which increased pulmonary vascular resistance (PVR) leads to right ventricular dysfunction and failure, and to premature death (1). Sustained pulmonary vasoconstriction and excessive pulmonary vascular remodeling, characterized by concentric pulmonary arterial wall thickening and occlusive intimal and plexiform lesions in the distal pulmonary artery (PA), are the 2 major causes for elevated PVR in patients with idiopathic and associated PAH and in animals with experimental pulmonary hypertension (PH) 2, 3, 4, 5. The proximal and distal PA wall is histologically composed of 3 layers of structure separated by elastic lamina: the adventitia formed mainly by fibroblasts and extracellular matrix, the media formed mainly by smooth muscle cells, and the thin intimal mainly formed by endothelial cells (ECs) (6). Although pulmonary arterial smooth muscle cell (PASMC) contraction is the primary cause for vasoconstriction, increased PASMC proliferation and migration are implicated in the development and progression of concentric PA wall thickening and arteriole and precapillary muscularization 7, 8. Multiple mechanisms and numerous intracellular signaling cascades are involved in stimulating PASMC proliferation and migration to develop pulmonary vascular remodeling 2, 9, 10, 11, 12, 13, 14, 15.

The phosphoinositide 3-kinase (PI3K)/AKT/mammalian target of rapamycin (mTOR) pathway, one of the critical signaling cascades involved in cell proliferation (16), can be activated by various growth factors and mitogenic cytokines 17, 18, 19. We and other investigators have shown that the PI3K/Akt1/mTOR signaling pathway plays an important role in the regulation of PASMC proliferation and the development of PH 16, 20. Activation of the PI3K/AKT/mTOR pathway in PASMCs is through various stimuli such as platelet-derived growth factor (PDGF), endothelin-1 21, 22, stress, and hypoxia (23). Global knock-out (KO) of the *Akt1* gene (*Akt1*^−/−^), but not the *Akt2* gene (*Akt2*^−/−^), significantly inhibited the development of experimental PH in mice (16), whereas overexpression of the phosphatase and tensin homolog (PTEN), a negative regulator of the PI3K/AKT/mTOR pathway 24, 25, exerted a similar protective effect on experimental PH in PTEN transgenic mice (16).

The downstream signaling protein, mTOR, in the PI3K/AKT/mTOR pathway is a serine/threonine kinase that belongs to the PI3K-related kinase family (26). We previously reported that conditional and inducible KO of *mTOR* in smooth muscle cells almost completely inhibited the development of PH in mice (16). These data provide compelling evidence that the PI3K/AKT1/mTOR signaling pathway in PASMC plays an important role in the development of PH. Specifically targeting signaling proteins and kinases in the PI3K/AKT1/mTOR cascade may help develop novel therapeutic approaches for idiopathic and associated PAH, as well as PH associated with lung diseases and hypoxia.

mTOR is a downstream signaling protein and a serine/threonine kinase of AKT1. mTOR is also a major kinase present in 2 functionally distinct complexes: the mTOR complex 1 (mTORC1) and the mTOR complex 2 (mTORC2) (27). mTORC1 is composed of mTOR, Raptor (regulatory associated protein of mammalian target of rapamycin), Pras40, GβL, and DEPTOR, and is inhibited by rapamycin and KU 0063794; mTORC2 is composed of mTOR, Rictor (rapamycin insensitive companion of mammalian target of rapamycin), GβL, Sin1, PRR5/Protor-1, and DEPTOR (26), and is inhibited by KU 0063794 (28). The individual protein complexes of mTORC have different upstream and downstream regulators (26). mTORC1 is a master growth regulator that promotes cell proliferation in response to growth factors, extracellular nutrients, and amino acids; mTORC2 promotes cell survival by activating AKT, regulates cytoskeletal dynamics by activating protein kinase C alpha, and controls ion transport and cell growth via serum/glucocorticoid-inducible kinase 1 phosphorylation. Global deletion of mTOR would disrupt the function of both mTORC1 and mTORC2. A relatively new topic of research in the field of pulmonary vascular disease is to understand the individual or differential roles of mTORC1 and mTORC2 in PASMC proliferation and the development of PAH/PH. The mTORC1 and mTORC2 also differ in their sensitivity to rapamycin; that is, short-term treatment with rapamycin inhibits mTORC1, but long-term treatment with rapamycin can inhibit both mTORC1 and mTORC2 (29).

Significant research is being conducted in understanding the role of mTOR, as a common component in both mTORC1 and mTORC2, in the development of hypoxia-induced PH by promoting PASMC proliferation 30, 31. The aim of the present study was to examine whether mTORC1 and mTORC2 potentially play a differential role in the development of PH. We generated the following: 1) smooth muscle (SM)-specific *Raptor* KO mice (*Raptor*^SM−/−^) to inhibit mTORC1 function in PASMCs; and 2) SM-specific *Rictor* KO mice (*Rictor*^SM−/−^) to inhibit mTORC2 function in PASMCs. We then conducted a series of experiments in wild-type (WT) *Raptor*^SM−/−^ and *Rictor*^SM−/−^ mice using combined techniques of in vitro cell and molecular biology, and in vivo hemodynamic measurement in intact mice, to define whether mTORC1 and mTORC2 are differentially involved in the development of PH and whether inhibition of mTORC1 and mTORC2 exerts the same therapeutic effect on experimental PH.

## Methods

A more detailed Methods and Materials section for this study is given in the Supplemental Material. The animal experimental procedures were approved by the Institutional Animal Care and Use Committee of The University of Arizona (Tucson, Arizona). All animals were bred and housed in the University of Arizona Animal Research Center. Standardized protocols were followed for conducting the experiments using rodent model.

## Results

We first conducted in vivo experiments using WT and various KO mice to examine whether SM-specific KO of mTOR (*mTOR*^SM−/−^), *Raptor* (*Raptor*^SM−/−^), and *Rictor* (*Rictor*^SM−/−^) exerted protective effects on experimental PH. In vitro Western blot experiments were then conducted by using PA isolated from WT and KO mice to examine whether functional disruption of mTORC1 in *mTOR*^SM−/−^ and *Raptor*^SM−/−^ mice or mTORC2 in *mTOR*^SM−/−^ and *Rictor*^SM−/−^ mice affects protein expression of platelet-derived growth factor receptor (PDGFR) α and PDGFRβ in PASMCs. Finally, we examined and compared the level of phosphorylated AKT (pAKT), a downstream signaling protein of mTORC2 and an upstream signaling protein of mTORC1, in PA isolated from WT and KO mice and in PASMCs isolated from normal subjects and patients with idiopathic PAH.

### Conditional and inducible KO of mTOR in PASMCs significantly inhibits the development of experimental PH

**Figure 1:**
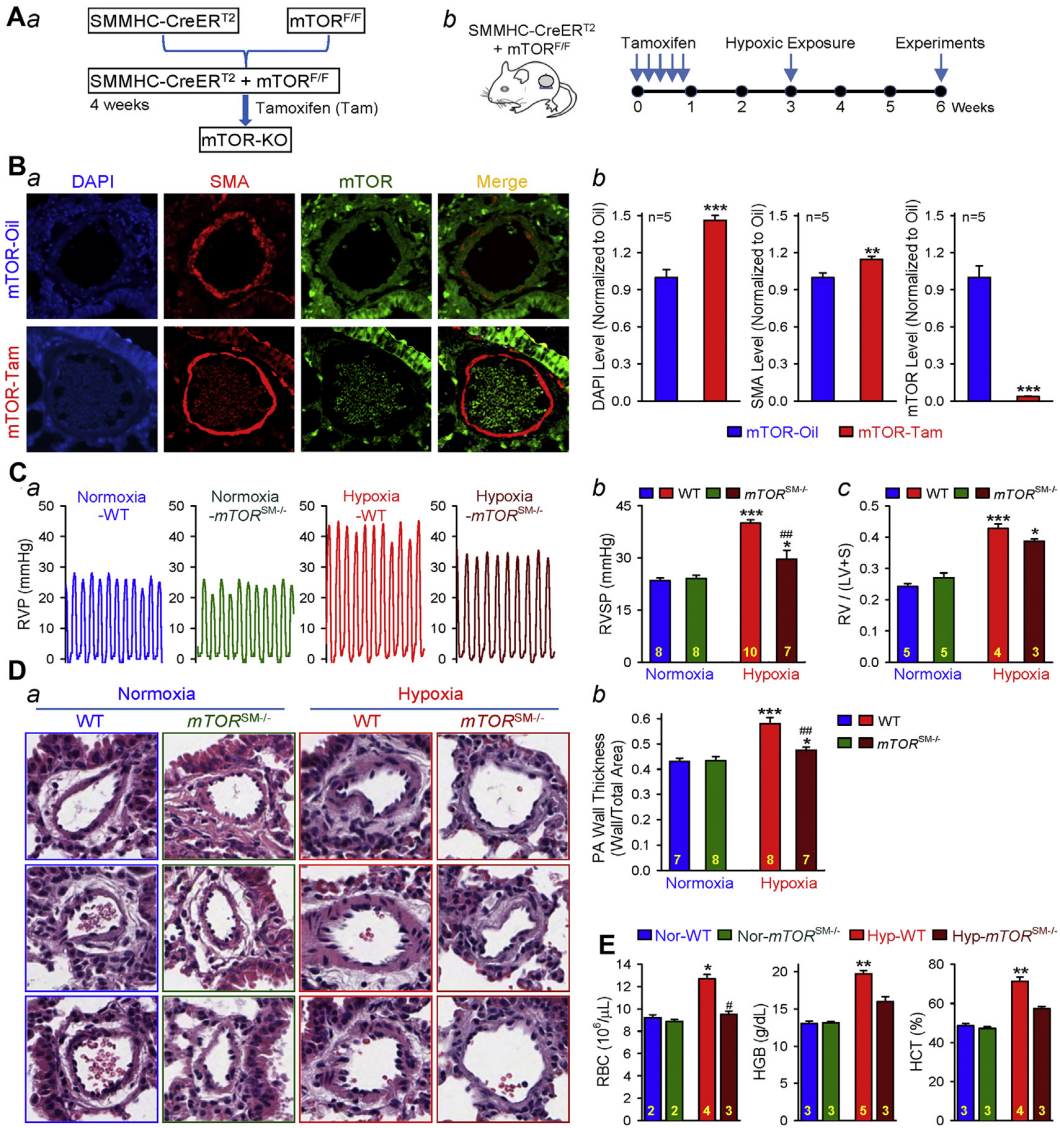
mTOR (mTORC1/mTORC2) in Smooth Muscle Cells and Pulmonary Hypertension Smooth muscle (SM)-specific conditional and inducible knock-out (KO) of *mTOR* attenuates hypoxia-induced pulmonary hypertension in *mTOR*^SM−/−^ mice. **(A)** Schematic strategy for the generation of *mTOR*^SM−/−^ mice **(*a*)** and the timeline indicating the time for injection of tamoxifen (Tam) (to induce *mTOR* KO), hypoxic exposure (for inducing pulmonary hypertension) and experimental measurements **(*b*)**. **(B)** Representative immunofluorescence images showing cell nuclei (4′,6′-diamidino-2-phenylindole [DAPI]; **blue**), smooth muscle cells (smooth muscle actin [SMA]; **red**), and mammalian target of rapamycin (mTOR; **green**) in the cross-section of small pulmonary artery (PA) in lung tissues from wild-type (WT) (mTOR-Oil) and *mTOR*^SM−/−^ (mTOR-Tam) mice **(*a*)**. Summarized data (mean ± SE; n = 5 in each group) for DAPI, SMA, and mTOR fluorescence intensity are shown in panels ***b***. It is noted that the mTOR **(green)** expression is almost abolished in the SMA-positive PA wall in mTOR-Tam mice but preserved in the mTOR-Oil mice. Student’s *t*-test (DAPI and SMA level) and Welch’s *t*-test (mTOR level), **p < 0.01 and ***p < 0.001 versus mTOR-Oil. **(C)** Representative record of right ventricular pressure (RVP) in WT and *mTOR*^SM−/−^ mice exposed to normoxia (room air, 21% oxygen) and hypoxia (10% oxygen for 3 weeks) **(*a*)**. Summarized data (mean ± SE) showing the peak value of right ventricular systolic pressure (RVSP) **(*b*)** (Kruskal-Wallis test, p < 0.001) and the Fulton index (the ratio of weight of the right ventricle divided by weight of the left ventricle plus the septum [RV/(LV + S)]) **(*c*)** (Kruskal-Wallis test, p = 0.005) in WT and *mTOR*^SM−/−^ mice exposed to normoxia and hypoxia. Dunn test, *p < 0.05, ***p < 0.001 versus Normoxia-WT; ^##^p < 0.01 versus Hypoxia-WT. **(D)** Representative hematoxylin and eosin images **(*a*)** of the cross-section of small PA and summarized data (mean ± SE) **(*b*)** showing the PA wall thickness in WT and *mTOR*^SM−/−^ mice under normoxic and hypoxic conditions. Kruskal-Wallis test, p < 0.001; Dunn test, **p < 0.01, *p < 0.05 versus Normoxia-WT, ^##^p < 0.01 versus Hypoxia-WT. **(E)** Summarized data (mean ± SE) showing the number of red blood cells (RBC) (Kruskal-Wallis test, p = 0.04), hemoglobin concentration (HGB) (Kruskal-Wallis test, p = 0.01), and hematocrit percentage (HCT) (Kruskal-Wallis test, p = 0.01) in WT and *mTOR*^SM−/−^ mice exposed to normoxia and hypoxia. Analysis of variance, **p < 0.01, *p < 0.05 versus Normoxia-WT; ^#^p < 0.05 versus Hypoxia-WT. The numbers of experiments (n) for each group are indicated in each bar.

To examine the role of mTOR (which is required for the function of both mTORC1 and mTORC2) in PASMC proliferation and the development of hypoxia-induced pulmonary hypertension (HPH), we first generated the SM-specific *mTOR* conditional and inducible KO mice (*mTOR*^SM−/−^) (Figure 1Aa) through crossing the floxed *mTOR* mice with a transgenic mouse line expressing a fusion protein of the Cre recombinase with the modified estrogen receptor binding domain (CreERT2) under the control of the SM myosin heavy chain promoter. To induce the KO of *mTOR*, we treated the Cre^+^mTOR^F/F^ mice with tamoxifen 5 times consecutively and waited for 1 to 2 weeks prior to exposing the mice to hypoxia (for 3 weeks) (Figure 1Ab). The control mice were treated with vehicle (Oil). Immunohistochemical staining of the PA in lung tissues showed that mTOR was expressed in all cell types, including PASMCs, in Cre^+^mTOR^F/F^ mice treated with the vehicle (Oil), whereas the mTOR expression was hardly detected in PASMCs or in PA in *mTOR*^SM−/−^ mice after treatment with tamoxifen (Figure 1B).

Deletion of mTOR would disrupt the function of both mTORC1 and mTORC2 (32). In this study, we repeated the experiments (Figure 1C) showing that SM-specific deletion of mTOR significantly inhibited the development of HPH. Chronic hypoxia-mediated increases in right ventricular systolic pressure (RVSP) (Figures 1Ca and 1Cb) in Cre^+^/*mTOR*^F/F^ mice treated with vehicle Oil (WT) was significantly attenuated compared with Cre^+^/*mTOR*^F/F^ mice treated with tamoxifen (*mTOR*^SM−/−^). The reduced RVSP was associated with the down-regulation of mTOR protein expression level in PASMCs (Figure 1Bb).

In addition to the change in RVSP, the hypoxia-induced pulmonary vascular wall thickening, determined by the PA wall thickness, was also significantly inhibited in *mTOR*^SM−/−^ mice compared with WT mice (Figure 1D). Hypoxia also resulted in polycythemia, indicated by the increased number of red blood cells, hemoglobin concentration (grams per deciliter) and hematocrit percentage (Figure 1E). It is noted that the hypoxia-induced polycythemia effect on red blood cell count and hemoglobin was also significantly inhibited in *mTOR*^SM−/−^ mice compared with WT mice. These data, consistent with our previous study (16), indicate that SM-specific deletion of *mTOR*, a serine/threonine kinase that is pivotal for the function of both mTORC1 and mTORC2, significantly inhibits the development of pulmonary arterial remodeling and HPH. The next set of experiments was designed to investigate whether mTORC1 and mTORC2 complexes are differentially involved in HPH.

### Conditional and inducible deletion of raptor in PASMCs significantly inhibits the development of experimental PH

**Figure 2:**
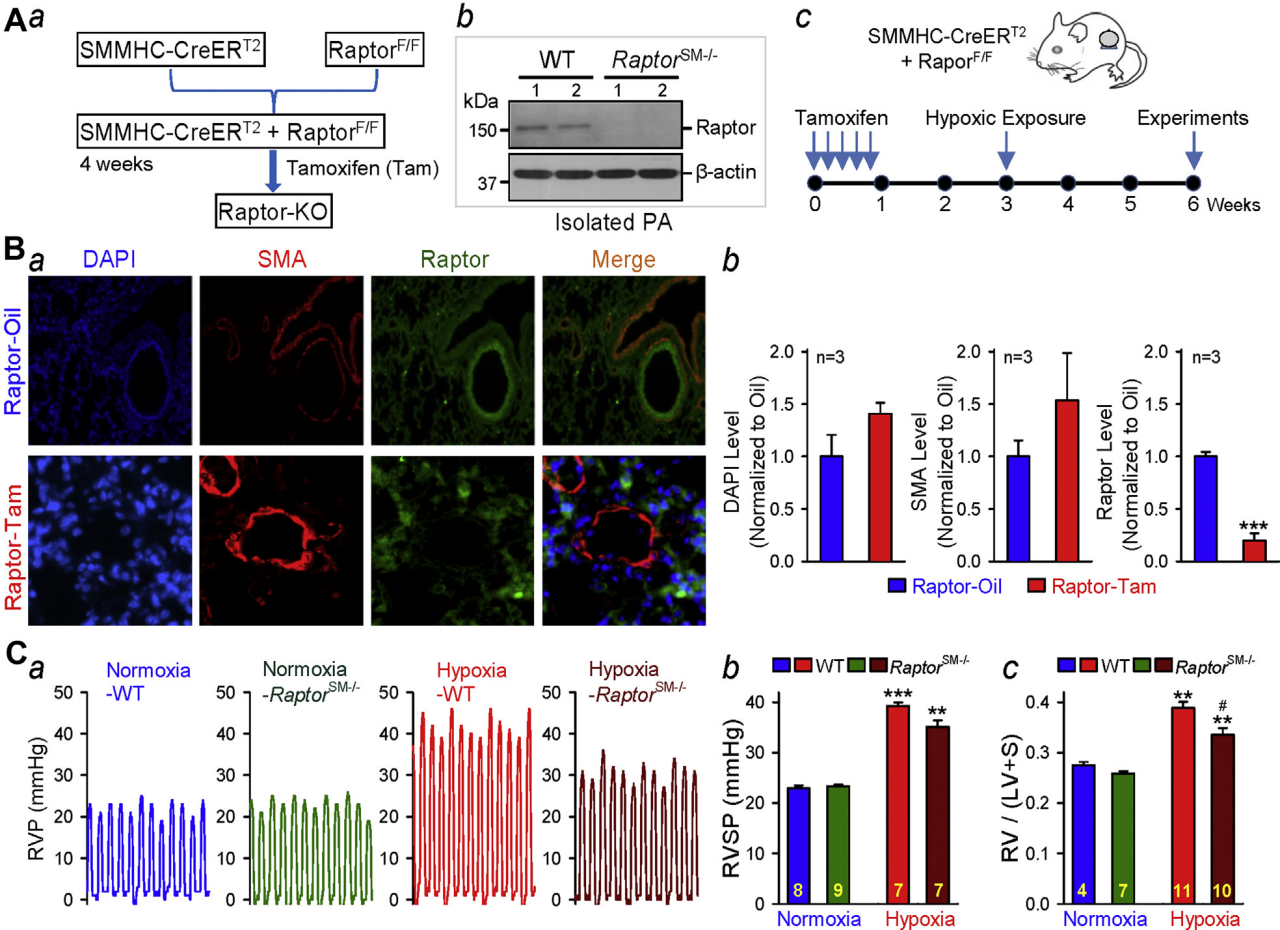
Raptor (mTORC1) in Smooth Muscle Cells and Pulmonary Hypertension SM-specific conditional and inducible KO of *Raptor* attenuates hypoxia-induced pulmonary hypertension in *Raptor*^SM−/−^ mice. **(A)** Schematic strategy for the generation of *Raptor*^SM–/–^ mice **(*a*)**; Western blot analysis of Raptor (regulatory associated protein of mammalian target of rapamycin) in isolated PA from WT and *Raptor*^SM−/−^ mice **(*b*)**; and the timeline indicating the time for Tam injection (to induce *Raptor* KO), hypoxic exposure (to induce PH), and experimental measurements **(*c*)**. **(B)** Representative immunofluorescence images showing cell nuclei (DAPI; **blue**), smooth muscle cells (SMA; **red**), and Raptor **(dark green)** in the cross-section of small PA in lung tissues from WT (Raptor-Oil) and *Raptor*^SM−/−^ (Raptor-Tam) mice **(*a*)**. Summarized data (mean ± SE; n = 3 in each group) for DAPI, SMA, and Raptor fluorescence intensity are shown in ***b***. Student’s *t*-test, ***p < 0.001 versus Raptor-Oil. **(C)** Representative record of RVP **(*a*)** as well as summarized data (mean ± SE) showing the peak value of RVSP **(*b*)** (Kruskal-Wallis test, p < 0.001) and the Fulton index (RV/[LV + S]) ratio **(*c*)** (Kruskal-Wallis test, p < 0.001) in WT (Oil-Cre^+^/*Raptor*^F/F^) and *Raptor*^SM−/−^ (Tam-Cre^+^/*Raptor*^F/F^) mice exposed to normoxia (room air, 21% oxygen) and hypoxia (10% oxygen for 3 weeks). Dunn test, ***p < 0.001, **p < 0.01 versus Normoxia-WT; #p < 0.05 versus Hypoxia WT. The numbers of experiments (*n*) for each group are indicated in each bar. Abbreviations as in Figure 1.

To examine the role of Raptor, which is responsible for the function of mTORC1, in PASMC proliferation, pulmonary vascular remodeling, and the development of PH, we generated SM-specific conditional and inducible *Raptor* KO mice (Figure 2Aa) using the same strategy as we used for generating *mTOR*^SM−/−^. Raptor deficiency in tamoxifen-treated Cre^+^Raptor^F/F^ mice was verified by Western blot analysis of pulmonary arterial vascular wall lysates from the respective mice (Figure 2Ab). To induce the KO of *Raptor*, we treated the Cre^+^Raptor^F/F^ mice with tamoxifen and waited for 1 to 2 weeks prior to exposure of the mice to hypoxia (for 3 weeks) for experiments (Figure 2Ac).

Raptor is expressed in PASMCs or PA in control mice (Figure 2Ba), whereas Raptor expression is significantly decreased in *Raptor*^SM−/−^ mice (Figure 2Bb, right panel). To examine the role of Raptor or mTORC1 in the development of experimental PH, we measured and compared the pulmonary hemodynamic variables (e.g., RVSP), right ventricular hypertrophy (RVH) (e.g., Fulton index) and pulmonary vascular remodeling (e.g., PA wall thickness) in *Raptor*^SM−/−^ and WT mice before and after exposure to hypoxia for 3 weeks. No significant difference in the basal pulmonary hemodynamic variables (e.g., RVSP) was observed between WT and *Raptor*^SM−/−^ mice under normoxic conditions (Figures 2Ca and 2Cb). Exposure of WT mice to normobaric hypoxia (10% O_2_) for 3 weeks resulted in significant increases in RVSP (from 23.5 ± 0.05 mm Hg to 39.8 ± 0.12 mm Hg; p < 0.001) (Figures 2Ca and 2Cb) and in the Fulton index (the ratio of weight of the right ventricle divided by weight of the left ventricle plus the septum [RV/(LV + S)] (from 0.28 ± 0.01 to 0.42 ± 0.07) (Figure 2Cc). In *Raptor*^SM−/−^ mice, the hypoxia-mediated increases in RVSP were slightly attenuated, but not statistically significant, and the RV/(LV + S) ratio was significantly attenuated compared with that in WT mice. These data indicate that SM-specific deletion of *Raptor* or SM-specific disruption of mTORC1 kinase activity exerts partial protective effects on experimental PH.

### Conditional and inducible deletion of rictor increases basal RVSP and negligibly affects the development of HPH

**Figure 3:**
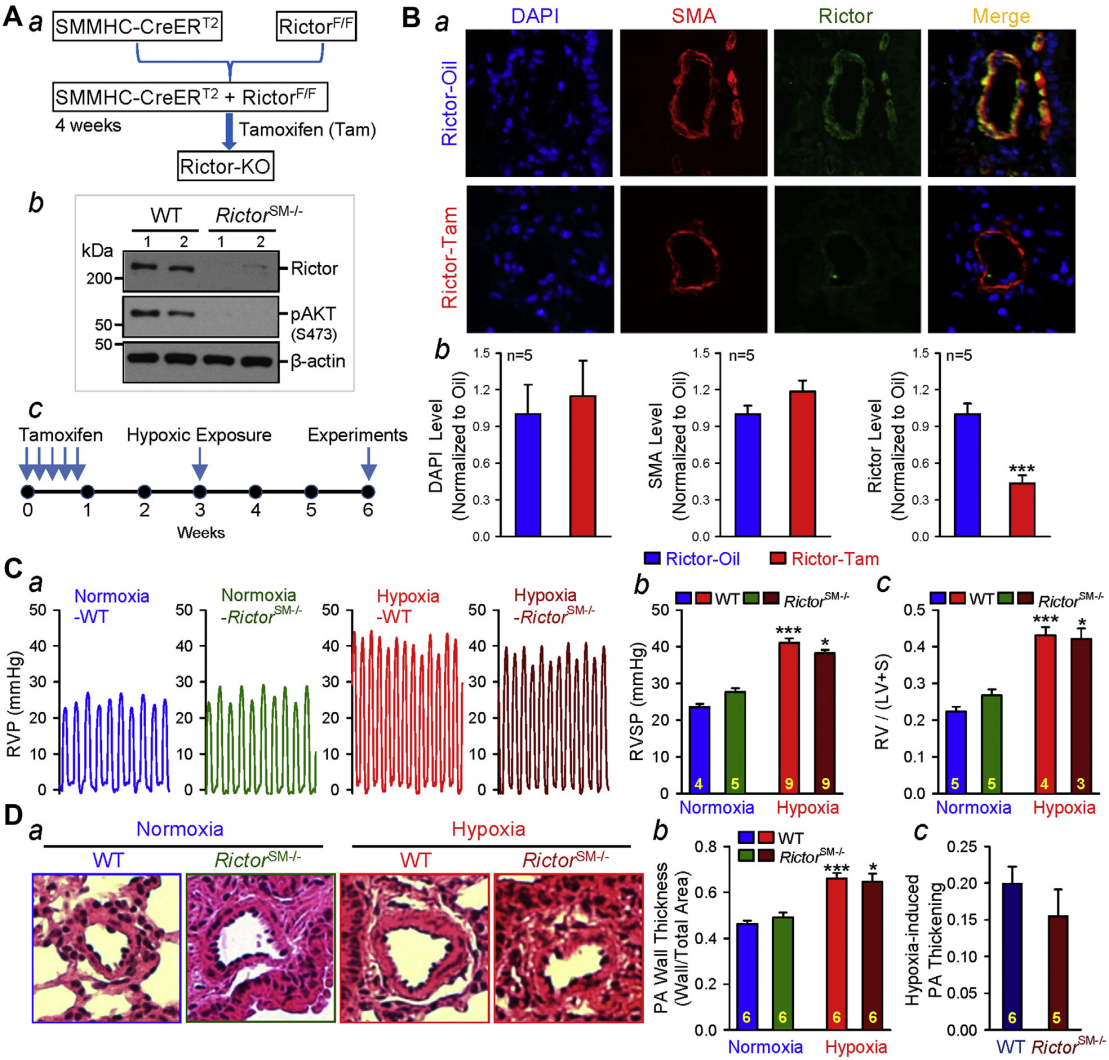
Rictor (mTORC2) in Smooth Muscle Cells and Pulmonary Hypertension SM-specific conditional and inducible KO of *Rictor* increases basal RVSP and negligibly affects the development of hypoxia-induced pulmonary hypertension in *Rictor*^SM−/−^ mice. **(A)** Schematic strategy for the generation of *Rictor*^SM−/−^ mice **(*a*)**; Western blot analysis of Rictor (rapamycin insensitive companion of mammalian target of rapamycin) in isolated PA from WT and *Rictor*^SM−/−^ mice **(*b*)**; and the timeline indicating the time for Tam injection (to induce *Rictor* KO), hypoxic exposure (to induce PH) and experimental measurements **(*c*)**. **(B)** Representative immunofluorescence images **(*a*)** and summarized data (mean ± SE; n = 5 in each group) **(*b*)** showing cell nuclei (DAPI; **blue**), smooth muscle cells (SMA; **red**), and Rictor **(dark green)** in the cross-section of small PA in lung tissues from WT (Rictor-Oil) and *Rictor*^SM−/−^ (Rictor-Tam) mice. Student’s *t*-test, ***p < 0.001 versus Rictor-Oil. **(C)** Representative record of RVP **(*a*)** and summarized data (mean ± SE) showing the peak value of RVSP **(*b*)** (Kruskal-Wallis test, p < 0.001) and the Fulton index (RV/[LV + S]) ratio **(*c*)** (Kruskal-Wallis test, p < 0.001) in WT and *Rictor*^SM−/−^ mice exposed to normoxia and hypoxia (for 3 weeks). Dunn test, *p < 0.05, ***p < 0.001 versus Normoxia-WT. **(D)** Representative hematoxylin and eosin images of the cross-section of small PA **(*a*)** and summarized data (mean ± SE) showing the PA wall thickness in WT and *Rictor*^SM−/−^ mice under normoxic and hypoxic conditions **(*b*)**. ***(c)*** Hypoxia-induced increase in PA wall thickness in WT and *Rictor*^SM−/−^ mice. The numbers of experiments (n) for each group are indicated in each bar. Abbreviations as in Figure 1.

It has been reported that mTORC2 has different physiological functions compared with mTORC1 20, 33, 34, and the functions and regulatory mechanisms of mTORC2 are less characterized and studied, especially in smooth muscle cells or PASMCs. To understand the potential role of Rictor or mTORC2 in the development of PH, we generated the SM-specific conditional and inducible *Rictor* KO mice (*Rictor*^SM−/−^) (Figure 3Aa). Rictor deficiency in *Rictor*^SM−/−^ mice was verified by Western blot analysis of pulmonary arterial vascular wall lysates from the respective mice (Figure 3Ab). To induce the KO of *Rictor*, we treated the Cre^+^Rictor^F/F^ mice with tamoxifen and waited for 1 to 2 weeks prior to the proposed hypoxic experiments (for 3 weeks) (Figure 1Ac). Immunohistochemistry experiments were conducted to confirm that the expression of Rictor was significantly down-regulated in PA cross-sections from the lung tissues in *Rictor*^SM−/−^ mice compared with WT mice (Figures 3Ba and 3Bb).

In contrast to *mTOR*^SM−/−^ and *Raptor*^SM−/−^ mice, the *Rictor*^SM−/−^ mice exhibited a slight, but not statistically significant, increase in RVSP and RVH (determined by using the Fulton index) under normoxic control conditions (Figures 3Ca and 3Cb) compared with WT mice. After 3 weeks of exposure to normobaric hypoxia, RVSP and RVH were further increased in both WT and *Rictor*^SM−/−^ mice; however, the hypoxia-induced increases in RVSP and RVH (Figure 3Cc) were comparable between WT and *Rictor*^SM−/−^ mice. It was noted that the nonsignificant difference in RVSP and the Fulton index between hypoxic WT mice and hypoxic *Rictor*^SM−/−^ mice was somehow related to a slight, but not statistically significant, increase in basal level of RVSP and RVH in *Rictor*^SM−/−^ mice (Figures 3Cb and 3Cc). These results indicate the following: 1) that SM-specific deletion of *Rictor* seems to, spontaneously, increase RVSP and RVH under normoxic control conditions; and 2) that SM-specific deletion of *Rictor* still inhibits hypoxia-induced PH.

To examine whether the hemodynamic data are consistent with the histological data, we also measured and compared PA wall thickness in lung tissues from WT and *Rictor*^SM–/–^ mice. The hypoxia-induced increases in RVSP and RVH in WT mice (Figure 3C) were associated with significant PA remodeling, indicated by a significant increase in wall thickness in small (<100 μm in diameter) arteries (Figures 3Da and 3Db). Similar to WT mice, *Rictor*^SM–/–^ mice also exhibited significant increases in PA wall thickness after 3 weeks of hypoxic exposure (Figure 3Dc); SM-specific deletion of *Rictor* failed to inhibit hypoxia-induced PA thickening (Figure 3Dd) during the development of HPH.

### Comparison of the changes in RVSP and the fulton index in conditional and inducible *mTOR*-KO *(mTOR*^SM−/−^)*, Raptor*-KO *(Raptor*^SM−/−^*),* and *Rictor*-KO *(Raptor*^SM−/−^*)* mice

By comparing the differences in RVSP and the Fulton index under normoxic and hypoxic conditions between *mTOR*^SM−/−^, *Raptor*^SM−/−^, and *Rictor*^SM−/−^ mice and their WT mice, we found that there was a slight, but not statistically significant, increase in both RVSP and the Fulton index in *Rictor*^SM−/−^ mice (∼3 weeks after injection of tamoxifen) under normoxic conditions compared with the WT littermates; however, there was no increase in RVSP or the Fulton index in *mTOR*^SM−/−^ and *Raptor*^SM−/−^ mice under normoxic conditions. The *mTOR*^SM−/−^ mice exhibited a 66% inhibition of hypoxia-induced increase in RVSP (from 16.5 ± 2.3 mm Hg in WT mice to 5.6 ± 2.5 mm Hg in *mTOR*^SM−/−^ mice); the *Raptor*^SM−/−^ mice exhibited a 28% inhibition of hypoxia-induced increase in RVSP (from 16.4 ± 1.5 mm Hg in WT mice to 11.8 ± 1.2 mm Hg in *Raptor*^SM−/−^ mice); and the *Rictor*^SM−/−^ mice exhibited a 39% inhibition of hypoxia-induced increase in RVSP (from 17.4 ± 1.7 mm Hg in WT mice to 10.6 ± 2.4 mm Hg in *Rictor*^SM−/−^ mice). Similar to the data on RVSP, the *mTOR*^SM−/−^ mice exhibited a 37% inhibition of hypoxia-induced increase in the Fulton index (from 0.20 ± 0.02 in WT mice to 0.12 ± 0.03 in *mTOR*^SM−/−^ mice); the *Raptor*^SM−/−^ mice exhibited a 36% inhibition of hypoxia-induced increase in the Fulton index (from 0.12 ± 0.12 in WT mice to 0.08 ± 0.01 in *Raptor*^SM−/−^ mice); and the *Rictor*^SM−/−^ mice exhibited a 27% inhibition of hypoxia-induced increase in the Fulton index (from 0.21 ± 0.03 in WT mice to 0.15 ± 0.04 mm Hg in *Rictor*^SM−/−^ mice).

### *Rictor-KO (Rictor*^SM−/−^*)* mice exhibit spontaneous PH due to pulmonary vascular remodeling

**Figure 4:**
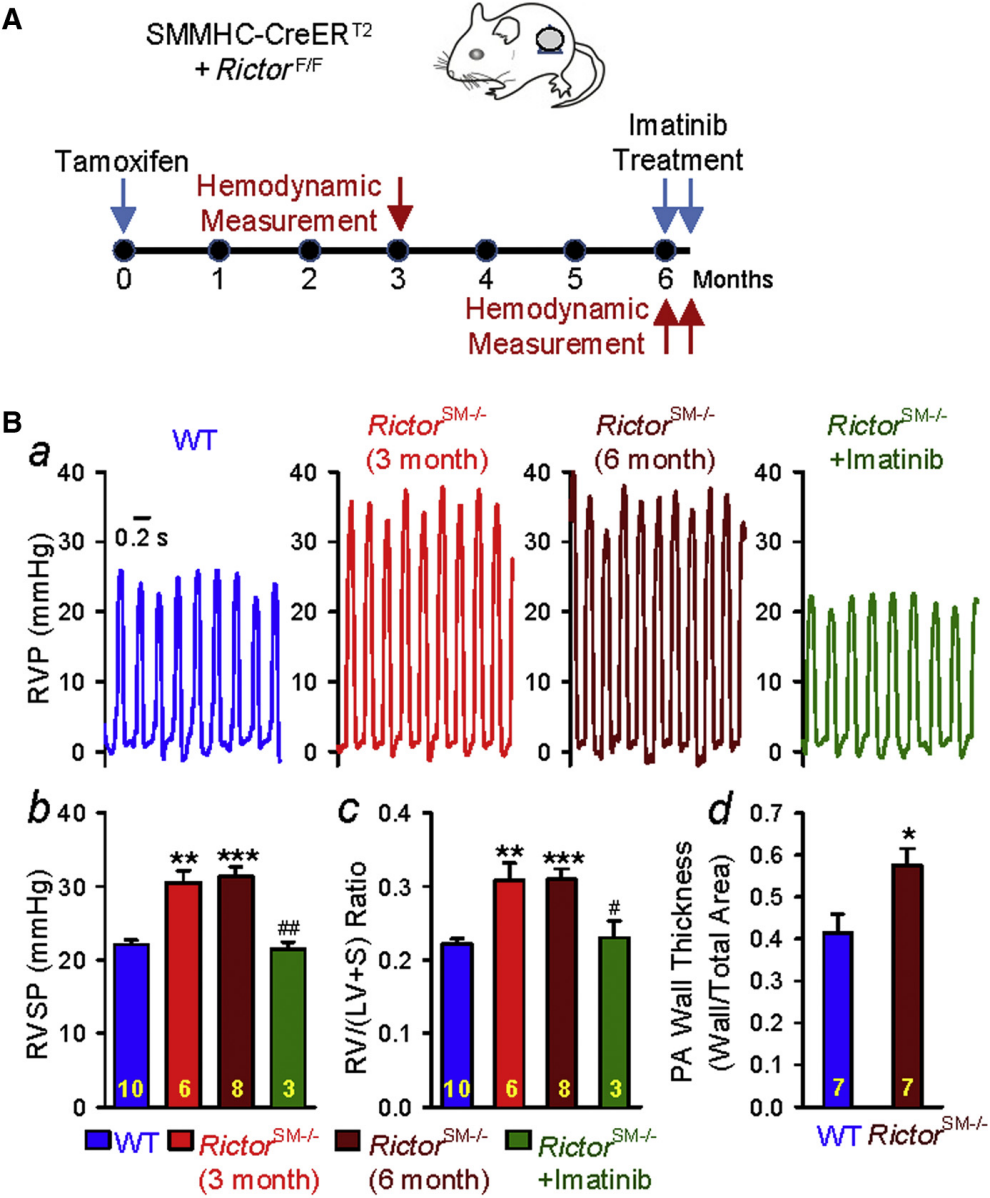
Rictor Deletion in Smooth Muscle Cells Causes Spontaneous Pulmonary Hypertension *Rictor*^SM−/−^ mice spontaneously develop PH and imatinib reverses the established PH in *Rictor*^SM−/−^ mice. **(A)** Schematic strategy for the generation of *Rictor*^SM−/−^ mice and the timeline indicating the time for Tam injection (to induce *Rictor* KO) and experimental measurements. **(B)** Representative record of RVP **(*a*)** and summarized data (mean ± SE) showing the peak value of RVSP **(*b*)** (Kruskal-Wallis test, *p* < 0.001) and the Fulton index (RV/[LV + S]) ratio **(*c*)** (Kruskal-Wallis test, *p* = 0.001) in WT and *Rictor*^SM−/−^ mice 3 and 6 months after Tam injection with or without 2-week intraperitoneal injection of imatinib (20 mg/kg) daily. Dunn test, *p < 0.05, **p < 0.001, ***p < 0.001 versus WT; ^#^*p* < 0.05, ^##^*p* < 0.01 versus *Rictor*^SM−/−^ (3 months). Summarized data of PA wall thickness in WT and *Rictor*^SM−/−^ mice 6 months after Tam injection **(*d*)**. *p < 0.05 versus WT. The numbers of experiments or mice (n) for each group are indicated in each bar. Abbreviations as in Figure 1.

To further confirm that *Rictor*^SM−/−^ mice may spontaneously develop PH under normoxic conditions, we measured RVSP and the Fulton index in *Rictor*^SM−/−^ mice at different times (3 or 6 months) after tamoxifen injection (Figure 4A) and then examined whether intraperitoneal injection of imatinib, a tyrosine kinase inhibitor that inhibits PDGFRs with high affinity, had a reversal effect on established PH in *Rictor*^SM−/−^ mice. As shown in Figure 4B, *Rictor*^SM−/−^ mice exhibited a significantly higher RVSP (Figures 4Ba and 4Bb) and Fulton index (Figure 4Bc) 3 months after injection of tamoxifen under normoxic conditions than the WT littermates. The increased RVSP and Fulton index were maintained for up to 6 months after tamoxifen injection (Figure 4Ba and 4Bb). Furthermore, we also examined whether increased RVSP in *Rictor*^SM−/−^ mice was associated with significant pulmonary vascular remodeling. As shown in Figure 4C, after 6 months of tamoxifen-induced *Rictor* deletion, *Rictor*^SM−/−^ mice displayed significantly increased PA wall thickness (Figure 4Bc) compared with the WT littermates. Inhibition of PDGFRs with imatinib reversed PH (determined by increased RVSP) and RV hypertrophy (determined by increased Fulton Index) in *Rictor*^SM−/−^ mice (Figure 4B).

These data indicate that SM-specific deletion of the Rictor gene (*Rictor*^SM−/−^) has a unique paradoxical effect on the pulmonary vasculature or the pulmonary circulatory system; the *Rictor* KO results in a spontaneous increase in RVSP, likely due to pulmonary vascular remodeling under normoxia. KO of neither *mTOR* nor *Raptor* in smooth muscle cells or PASMC has the spontaneous augmenting effect on RVSP and PA wall thickening. The protective effect of mTOR-KO is greater than the effect of *Raptor*-KO or *Rictor*-KO, suggesting that both mTORC1 and mTORC2 are involved in the development of HPH.

### Endothelial-specific KO of Rictor fails to induce spontaneous pulmonary hypertension in *Rictor*^EC−/−^ mice

**Figure 5:**
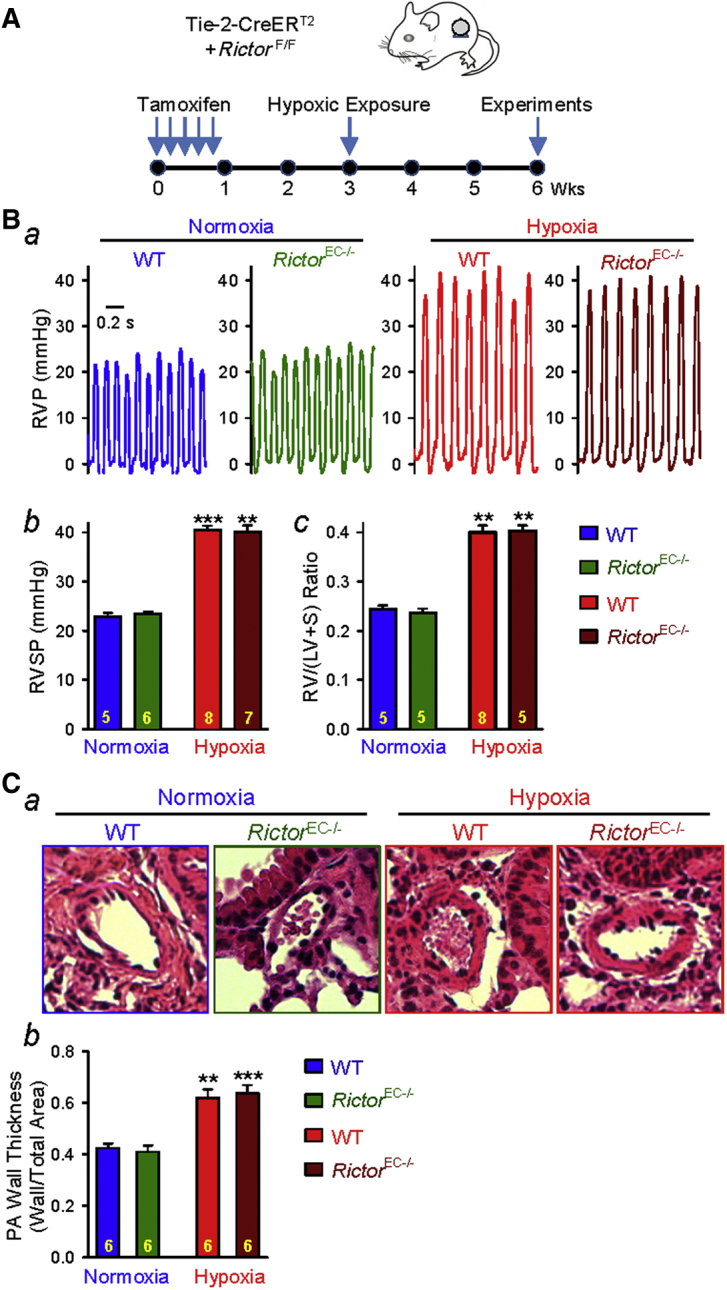
Rictor Deletion in Endothelial Cells Fails to Cause Spontaneous Pulmonary Hypertension Endothelial-specific conditional and inducible KO of *Rictor* fails to attenuates hypoxia-induced pulmonary hypertension in *Rictor*^EC−/−^ mice. **(A)** Schematic strategy for the generation of EC-specific *Rictor*-KO mice (*Rictor*^EC−/−^) and the timeline indicating the time for Tam injection and experimental measurements. **(B)** Representative record of RVP **(*a*)** and summarized data (mean ± SE) showing the peak value of RVSP **(*b*)** (Kruskal-Wallis test, p < 0.001) and the Fulton index (RV/[LV + S]) ratio **(*c*)** (Kruskal-Wallis test, p < 0.001) in WT and *Rictor*^EC−/−^ mice exposed to normoxia and hypoxia (for 3 weeks). Dunn test, **p < 0.01, ***p < 0.001 versus Normoxia-WT. **(C)** Representative hematoxylin and eosin images of the cross-section of small PA **(*a*)** and summarized data (mean ± SE) showing the PA wall thickness in WT and *Rictor*^EC−/−^ mice under normoxic and hypoxic conditions **(*b*)**. The numbers of experiments or samples (n) for each group are indicated in each bar. Abbreviations as in Figure 1.

To confirm that the pathogenic role of *Rictor* KO or mTORC2 inhibition is specific to smooth muscle cells, we created an EC-conditional KO mouse strain by crossing floxed mice with Tie2-CreER mice in which Cre expression is under the control of Tie2 promoter (Figure 5A). Similar hemodynamic and histological experiments were then conducted in the EC-specific *Rictor* KO (*Rictor*^EC−/−^) mice. In contrast to the findings from the SM-specific KO mice (*Rictor*^SM−/−^), we found that neither the basal RVSP (Figures 5Ba and 5Bb) and Fulton index (Figure 5Bc) nor the hypoxia-induced increases in RVSP and the Fulton index were changed in *Rictor*^EC-/-^ mice compared with the WT littermates. Furthermore, both WT and *Rictor*^EC−/−^ mice exhibited significant increases in pulmonary vascular remodeling, determined according to PA wall thickness, after 3 weeks of hypoxic exposure; no difference was observed between WT mice and *Rictor*^EC−/−^ mice (Figures 5Ca and 5Cb). These data suggest that endothelial-specific KO of *Rictor* has neither a protective effect on hypoxia-induced PH nor a pathogenic effect on pulmonary vascular remodeling to increase RVSP under normoxic conditions. The spontaneous PH and consistent pulmonary vascular remodeling in *Rictor*^SM−/−^ mice are specific to Rictor/mTORC2 inhibition in pulmonary vascular smooth muscle cells.

### Isolated PA or PASMCs from *Rictor*^*SM−/−*^ mice exhibit up-regulation of PDGFRs (PDGFRα and PDGFRβ)

**Figure 6:**
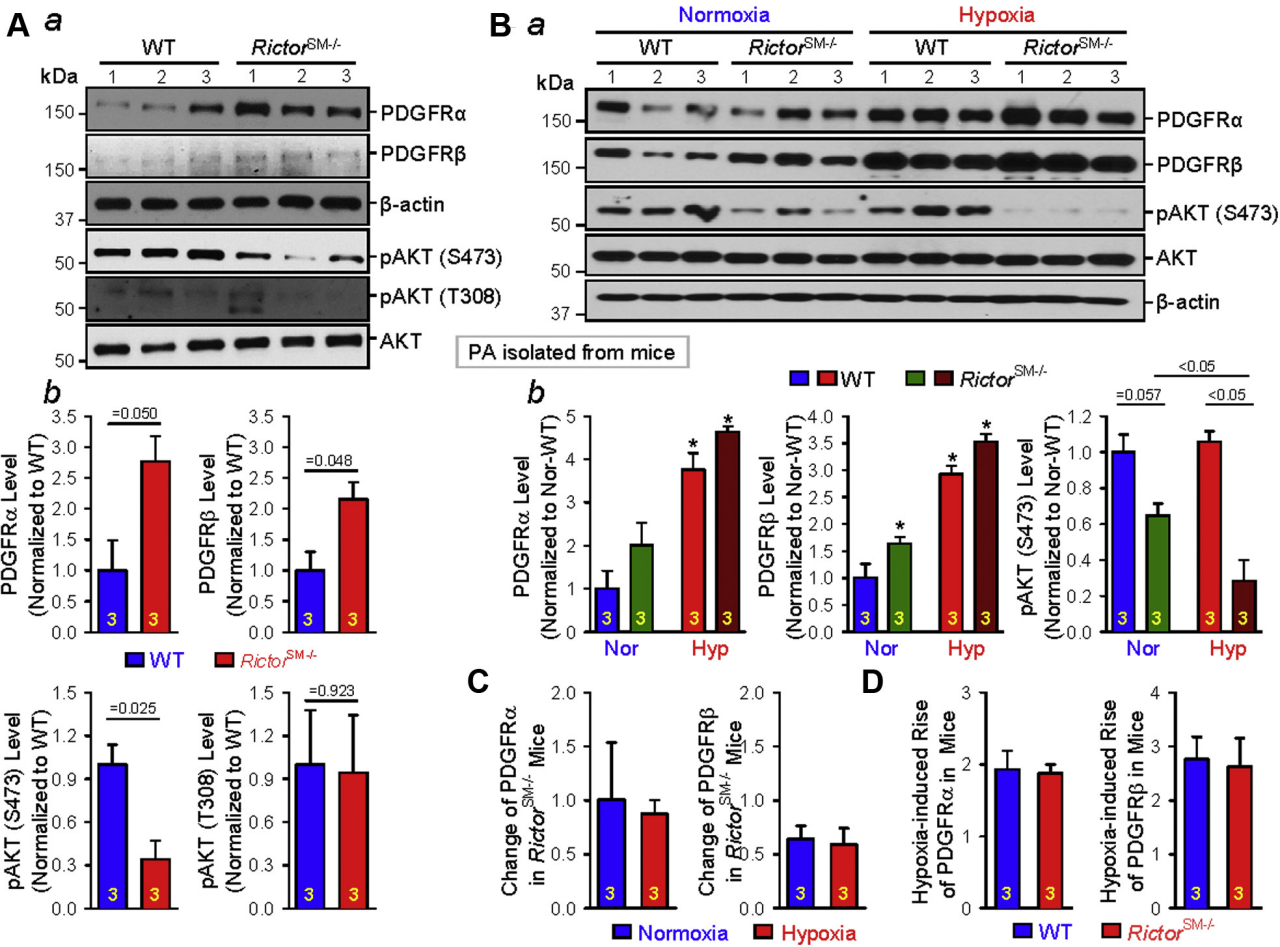
Rictor (mTORC2) Contributes to Regulating PDGFR Expression in Smooth Muscle Cells SM-specific conditional and inducible KO of *Rictor* up-regulates the protein expression of platelet-derived growth factor receptors (PDGFRs) in isolated PA. **(A)** Western blot analyses on PDGFRα and PDGFRβ, as well as phosphorylated AKT at S473 and T308, in PA isolated from WT and *Rictor*^SM−/−^ mice **(*a*)**. Summarized data (mean ± SE; n = 3 in each group) showing PA expression levels of PDGFRα, PDGFRβ, pAKT (S473), and pAKT (T308) in WT and *Rictor*^SM−/−^ mice in PA isolated from WT and *Rictor*^SM−/−^ mice **(*b*)**. *p* values, determined by Student's *t*-test are indicated in ***b***, WT vs. Rictor^SM−/−^ mice. **(B)** Western blot analyses on PDGFRα, PDGFRβ, pAKT (S473), and AKT in PA isolated from normoxic and chronically hypoxic WT and *Rictor*^SM−/−^ mice **(*a*)**. Summarized data (mean ± SE, n = 3 in each group) showing PA expression levels of PDGFRα, PDGFRβ, and pAKT (S473) in WT and *Rictor*^SM−/−^ mice under normoxic and hypoxic conditions **(*b*)**. Kruskal-Wallis test, p = 0.02 and Dunn test, *p < 0.05 versus Normoxia-WT or Normoxia-*Rictor*^SM−/−^. **(C)** Summarized data (mean ± SE; n = 3 in each group) showing the changes (or differences) in PA expression levels of PDGFRα **(left panel)** and PDGFRβ **(right panel)** in *Rictor*^SM−/−^ mice (compared with the WT controls) during normoxia and hypoxia. **(D)** Summarized data (mean ± SE; n = 3 in each group) showing the hypoxia-induced changes in the protein expression level of PDGFRα and PDGFRβ in WT mice and *Rictor*^SM−/−^ mice. The numbers of experiments (n) for each group are also indicated in each bar. Abbreviations as in Figure 1.

Increased PDGF and up-regulated PDGFRs have been implicated in the development and progression of idiopathic and associated PAH and PH associated with hypoxia and lung disease (35). To examine whether deletion of *Rictor* increases RVSP and the Fulton index due, at least partially, to up-regulation of PDGFRs in PA or PASMCs, we isolated PA from WT and *Rictor*^SM−/−^ mice and compared protein expression levels of PDGFRα and PDGFRβ. The expression level of PDGFRα and PDGFRβ were greater in PA isolated from *Rictor*^SM−/−^ mice than in PA isolated from WT mice (Figures 6Aa, b and 6Ba, b). The KO of *Rictor* would lead to inhibition of mTORC2. Indeed, pAKT (at S473 but not at T308), a major downstream signaling protein of mTORC2, was significantly decreased in the PA isolated from *Rictor*^SM−/−^ mice compared with the PA isolated from WT mice (Figures 6A and 6B). The total AKT protein expression level, however, did not differ in isolated PA between WT and *Rictor*^SM−/−^ mice (Figures 6Aa and 6Ba). These data indicate that SM-specific deletion of *Rictor* or inhibition of mTORC2 in *Rictor*^SM−/−^ mice results in a spontaneous up-regulation of PDGFRs (PDGFRα and PDGFRβ) in PASMCs. It is unclear, however, whether the up-regulated PDGFRα/PDGFRβ were related to decreased pAKT (at S473) in PA isolated from *Rictor*^SM−/−^ mice.

We next examined and compared the expression level of PDGFRα and PDGFRβ in PA isolated from WT and *Rictor*^SM−/−^ mice under normoxic and hypoxic conditions. Similar to the data shown in Figure 6A, both PDGFRα and PDGFRβ protein expression levels were increased in PA isolated from *Rictor*^SM−/−^ mice compared with the PA from WT mice. In PA isolated from WT mice with HPH, the protein expression level of PDGFRα and PDGFRβ was significantly greater than in normoxic control WT mice. SM-specific KO of *Rictor* in *Rictor*^SM−/−^ mice caused a slight, but not statistically significant, up-regulation of PDGFRα and PDGFRβ in PA under hypoxic conditions compared with their WT controls (Figure 6Bb, left and middle panels). Furthermore, SM-specific KO of *Rictor* significantly decreased pAKT at S473 in PA isolated from mice exposed to normoxia and hypoxia; the decreasing effect of *Rictor* KO on pAKT (S473) was enhanced under hypoxic conditions (Figure 6Bb, right panel). Quantitative analyses of the pulmonary arterial PDGFRα and PDGFRβ levels in WT and *Rictor*^SM−/−^ mice indicate that the up-regulation of PDGFRα and PDGFRβ (Figure 6C) in PA associated with SM-specific KO of *Rictor* under normoxic conditions is the same as that under hypoxic conditions. Furthermore, hypoxia-induced increases in PDGFRα/PDGFRβ protein expression in PA isolated from WT mice was not different from that in PA isolated from *Rictor*^SM−/−^ mice (Figure 6D). The decrease in pAKT (S473) due to SM-specific KO of *Rictor*, however, was significantly greater in hypoxia than in normoxia (Figure 6Bb, right panel). These data imply that *Rictor*-KO and hypoxia both up-regulate PDGFRα/PDGFRβ in PA; the mechanisms involved in hypoxia-induced PDGFRα/PDGFRβ up-regulation may be different from the mechanism by which *Rictor*-KO (or mTORC2 inhibition) increases PDGFRα/PDGFRβ expression.

### Pharmacological inhibition of mTORC2 up-regulates the expression of PDGFRα and PDGFRβ in human PASMCs

**Figure 7:**
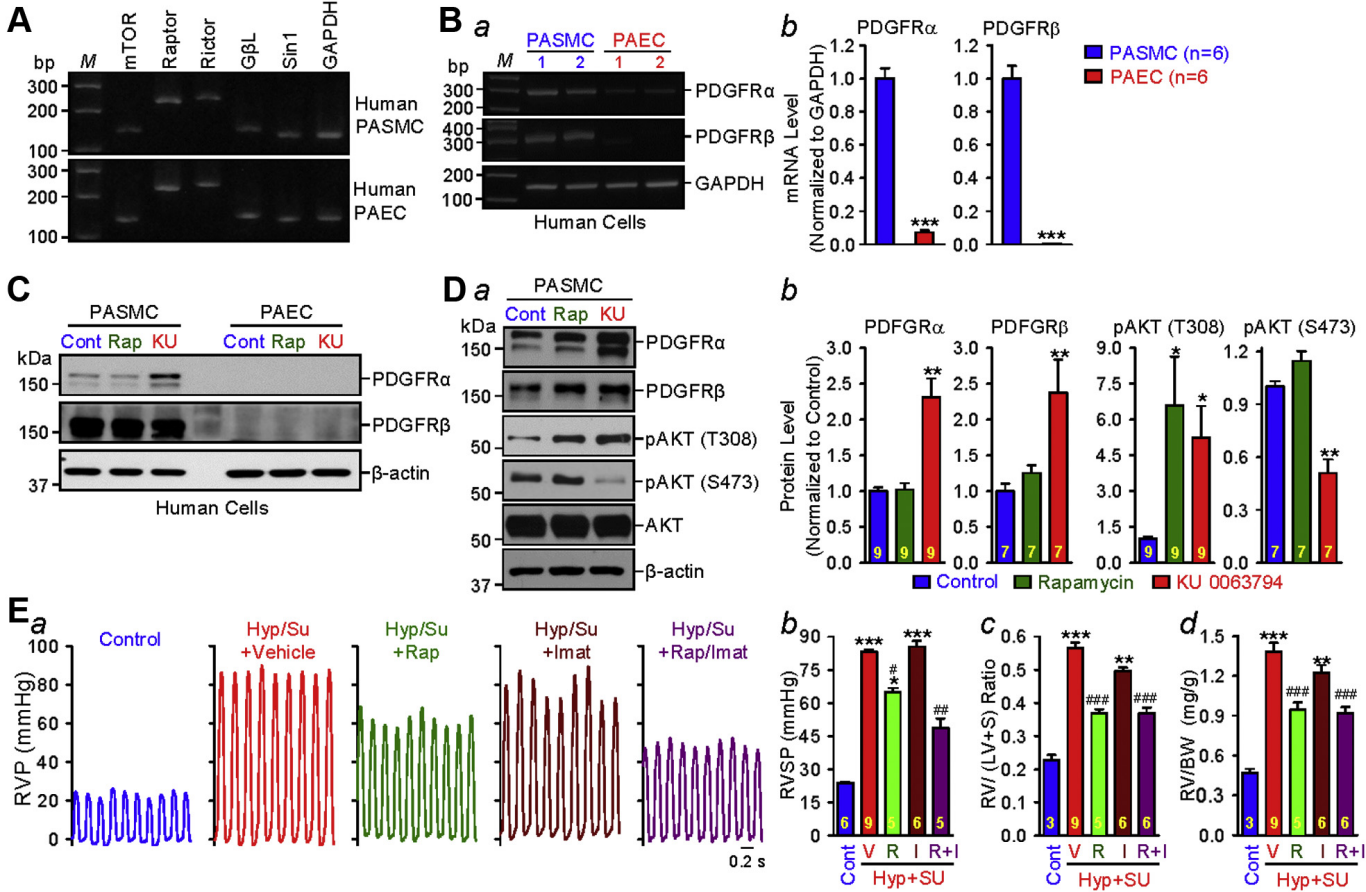
mTORC2 Regulates PDGFR Expression in Smooth Muscle Cells Pharmacological inhibition of mammalian target of rapamycin complex 2 (mTORC2) up-regulates the protein expression of PDGFRα and PDGFRβ in pulmonary arterial smooth muscle cells (PASMCs). Reverse transcription polymerase chain reaction analyses on **(A)** mTOR, Raptor, Rictor, GβL, and Sin1 as well as **(B)** PDGFRα and PDGFRβ **(*a*)** in human PASMCs and pulmonary arterial endothelial cells (PAECs). Summarized data (mean ± SE) **(*b*)** showing the levels of PDGFRα and PDGFRβ in human PASMCs (n = 6) and PAECs (n = 6). Welch’s *t-*test, ***p < 0.001 vs PASMC. **(C)** Western blot analyses on PDGFRα and PDGFRβ in human PASMCs and PAECs treated with vehicle (Cont), 50-nM rapamycin (Rap), and 200-nM KU 0063794 (KU) for 24 h. **(D)** Western blot analyses on PDGFRα, PDGFRβ, pAKT (T308), pAKT (S473), and AKT in PASMCs treated with vehicle (Cont), Rap, and KU (for 24 h) **(*a*)**. Summarized data (mean ± SE) showing the levels of PDGFRα (Kruskal-Wallis test, p < 0.001; *n* = 9), PDGFRβ (Kruskal-Wallis test, p = 0.01; *n* = 7), pAKT (T308) (Kruskal-Wallis test, p < 0.001; *n* = 9), and pAKT (S473) (Kruskal-Wallis test, p < 0.001; n = 7) in control PASMC and PASMC-treated Rap and KU **(*b*)**. Dunn test, *p < 0.05, **p < 0.01 versus Control. **(E)** Representative record of RVP in control rats and hypoxia/Sugen (Hyp/Su) rats treated with vehicle, Rap (5 mg/kg body weight, intraperitoneally), imatinib (Imat, 20 mg/kg body weight, intraperitoneally), or combination of Rap and Imat (Rap/Imat) **(*a*)**. Summarized data (mean ± SE) **(*b*–*d*)** in control rats (Cont) and Hyp/Su rats treated with vehicle (V), Rap (R), Imat (I), or Rap and Imat (R + I) showing peak RVSP **(*b*)** (Kruskal-Wallis test, p < 0.001), the Fulton index **(*c*)** (Kruskal-Wallis test, p < 0.001), and the ratio of right ventricle weight to body weight (RV/BW) **(*d*)** (Kruskal-Wallis test, p < 0.001). Dunn test, *p < 0.05, **p < 0.01, ***p < 0.001 versus Control; Student’s *t*-test, ^#^p < 0.05, ^##^p < 0.01, ^###^*p*<0.001 versus Hyp/Su with Vehicle (V) and Hyp/Su with Imatinib (I). Abbreviations as in Figures 1 and 6.

Increased proliferation of PASMCs and pulmonary arterial endothelial cells (PAECs) has been implicated in the development and progression of pulmonary vascular remodeling and PH (36). Our reverse transcription polymerase chain reaction experiments (Figure 7A) revealed the presence of messenger ribonucleic acid expression of mTOR, Raptor, Rictor, Gβl, and Sin1 in human PASMCs and PAECs, whereas Western blot experiments (Figures 7Ba and 7Bb) indicated that PDGFRα and PDGFRβ were both expressed in human PASMCs but not in human PAECs (or the expression level of PDGFRα and PDGFRβ in the PAECs was too low to be detected). To further examine whether mTORC2 is involved in up-regulating the expression of PDGFRα and PDGFRβ in vitro, PDGFRα and PDGFRβ expression levels were compared in human PASMCs treated with vehicle (Control), rapamycin (50 nM), or KU 0063794 (200 nM). Because we were unable to detect PDGFRs in PAECs (Figure 7B), the focus of the in vitro experiments was on PASMCs.

Short-term treatment (24 h) with rapamycin (50 nM), an mTOR1-specific inhibitor (although long-term treatment with rapamycin has been reported to inhibit mTOR2 as well) (37), had a negligible effect on the protein expression levels of PDGFRα and PDGFRβ in PASMCs, increased pAKT (T308), and had no effect on pAKT (S473) (Figures 7C and 7D). Treatment (24 h) with KU 0063794, an inhibitor for both mTORC1 and mTORC2, significantly up-regulated the protein expression of PDGFRα and PDGFRβ and increased pAKT (T308) but decreased pAKT (S473). Both rapamycin and KU 0063794 increased the activity of pAKT (T308), but only KU 0063794 decreased the activity of pAKT (S473). These in vitro experimental results indicate that inhibition of mTORC2 can up-regulate the expression of PDGFRα and PDGFRβ in human PASMCs, which is in agreement with our in vivo data in mice showing that SM-specific KO of *Rictor* (which disrupts mTORC2) up-regulates PDGFRα and PDGFRβ in PA and contributes to spontaneous increases in RVSP and the Fulton index in *Rictor*^SM−/−^ mice under normoxic control conditions.

Inhibition of mTORC2, either by SM-specific KO of *Rictor* in vivo or pharmacological blockade with KU 0063974 in vitro, up-regulates PDGFRα/PDGFRβ in PASMCs. Therefore, treatment of PH with duel inhibitors of mTORC1 and mTORC2, such as rapamycin, should be combined with a blocker of PDGFRs. To test this hypothesis, we conducted in vivo experiments to examine whether combination treatment with low doses of rapamycin and imatinib (an inhibitor of PDGFRs) would yield a better therapeutic effect on experimental PH. As shown in Figure 7E, intraperitoneal injection of a low dose of rapamycin (5 mg/kg) resulted in a 29% inhibition of hypoxia/Sugen–mediated increase in RVSP (Figures 7Ea and 7Eb) and RVH (determined by the ratio of RV/[LV + S] and the ratio of right ventricle weight to body weight) (Figures 7Ec and 7Ed). The low dose of imatinib (20 mg/kg) had no effect on RVSP (Figures 7Ea and 7Eb) but slightly (with statistical significance) inhibited hypoxia/Sugen –mediated increases in the Fulton index (Figure 7Ec) and the ratio of right ventricular weight to body weight (Figure 7Ed). However, combination of the low doses of rapamycin and imatinib resulted in ∼60% inhibition of the hypoxia/Sugen–mediated increase in RVSP (Figures 7Ea and 7Eb). These data suggest that blockade of PDGFRs using tyrosine kinase receptor antagonists (e.g., imatinib) could enhance the therapeutic effect of mTORC1/mTORC2 inhibitors.

The low dose of rapamycin alone, however, resulted in a 58.3% inhibition of the hypoxia/Sugen–induced increase in the Fulton index, while combination treatment of rapamycin and imatinib did not further inhibit the hypoxia/Sugen–mediated increase in the Fulton index (by 57.9%) (Figure 7Ec). Further study is needed to investigate the potential difference of the effect of rapamycin and imatinib on pulmonary vascular smooth muscle cells and cardiomyocytes.

### Pharmacological inhibition of mTORC2 increases the nuclear forkhead box O3 level in PASMCs

**Figure 8:**
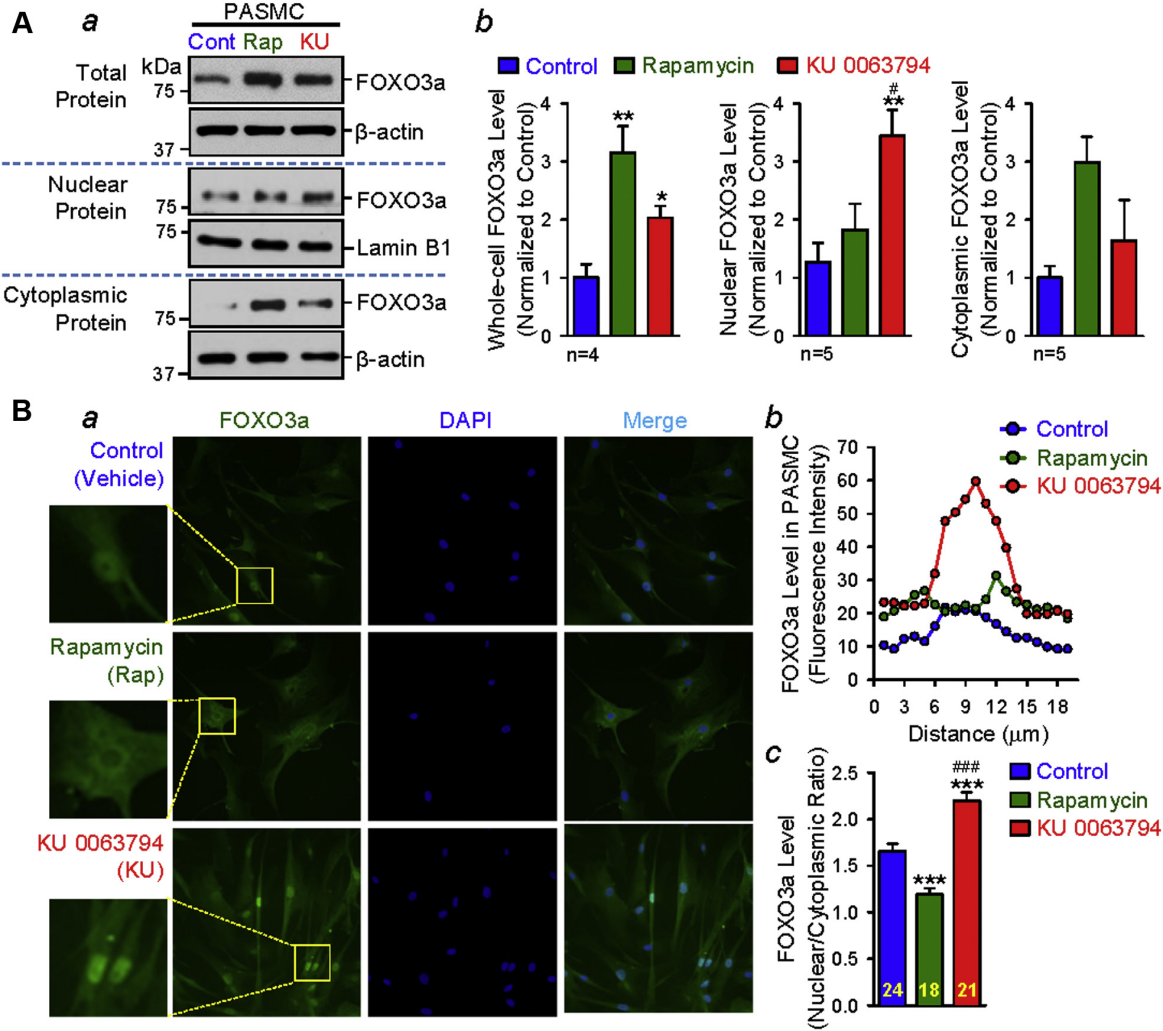
mTORC2 Regulates PDGFR Expression via FOXO3a in Smooth Muscle Cells Pharmacological inhibition of mTORC2 up-regulates the nuclear protein expression of Forkhead box O3a (FOXO3a) in human PASMCs. **(A)** Representative images showing Western blot analyses on FOXO3a in the total, nuclear, and cytoplasmic proteins idolated from human PASMCs treated (for 24 h) with vehicle (Cont), Rap (50 nM), and KU 0063794 (KU, 200 nM) **(*a*)**. Summarized data (mean ± SE) showing the total (Kruskal-Wallis test, p = 0.01; n = 4 in each group), nuclear (Kruskal-Wallis test, p = 0.01; n = 5 in each group), and cytoplasmic (Kruskal-Wallis test, p = 0.06; n = 5 in each group) protein levels of FOXO3a in PASMCs treated with vehicle (Control; **blue bars**), Rap (**green bars**), and KU (**red bars**) **(*b*)**. Dunn test, *p < 0.05, **p < 0.01 versus Control; #p < 0.05 versus rapamycin. **(B)** Representative immunofluorescence images showing cell nuclei (DAPI; **blue**) and FOXO3a **(dark green)** in human PASMCs treated (for 24 h) with vehicle (Control), Rap (50 nM), and KU (200 nM) **(*a*)**. The fluorescence intensity of FOXO3a along the line across a PASMC **(*b*)** treated with vehicle (Control), Rap, and KU. Summarized data (mean ± SE) showing the mean fluorescence intensity of the nuclear FOXO3a **(*c*)** in Control (n = 24), Rap-treated (n = 18), and KU-treated (n = 21) PASMCs; the value was calculated as the ratio of the nuclear intensity of FOXO3a to the cytoplasmic fluorescence intensity of FOXO3a. Kruskal-Wallis test, p < 0.001 and Dunn test, ***p < 0.001 versus Control; ^###^p < 0.001 versus rapamycin. Abbreviations as in Figures 1 and 7.

Forkhead box O3a (FOXO3a) is a transcription factor that promotes cell apoptosis and induces cell cycle arrest (38). FOXO3a is inhibited by AKT or by pAKT-mediated phosphorylation at T32, S253, and S315; more specifically, nuclear translocation of FOXO3a is inhibited by pAKT-mediated phosphorylation at T32, S253, and S315. Therefore, pAKT-associated phosphorylation of FOXO3a, due to aberrantly activated mTOC2/AKT 39, 40, leads to transcriptional inhibition of target genes such as the PDGFRs 41, 42. We found that treatment (24 h) of human PASMCs with rapamycin or KU 0063794 increased the level of total FOXO3a (or FOXO3a in the total protein of the PASMCs). Interestingly, the nucleocytoplasmic separation experiments showed that rapamycin seemed to have a slight, but not statistically significant, increasing effect on the protein level of cytoplasmic FOXO3a, whereas KU 0063794 mainly increased protein level of nuclear FOXO3a (Figure 8A). Furthermore, the immunohistochemistry experiments produced similar results. Both rapamycin and KU 0063794 increased the protein expression level of FOXO3a in human PASMCs (Figure 8B). The fluorescence intensity of FOXO3a (green) was mainly increased in the cytoplasm after rapamycin treatment, whereas the fluorescence intensity of FOXO3a (green) was mainly increased in the nuclei after KU 0063794 treatment (Figure 8Ba). The line-scan and bar graphs (Figures 8Bb and 8Bc) all show that KU 0063794 significantly decreased pAKT at S473 (Figure 7D) and increased the nuclear FOXO3a (Figures 8Bb and 8Bc), whereas rapamycin did not significantly decrease pAKT at S473 (Figure 7D) and actually decreased the nuclear FOXO3a (Figure 8Bc). Taken together, these data indicate that inhibition of mTORC2 increases the activity of FOXO3a in PASMCs, which may be the mechanism in which PDGFR is negatively regulated by mTORC2.

## Discussion

Increased PVR is a major contributor to the elevated pulmonary arterial pressure in patients with idiopathic PAH (43). Sustained pulmonary vasoconstriction (due to PASMC contraction) and concentric pulmonary vascular wall thickening (due partially to PASMC proliferation and migration) are 2 major causes of elevated PVR (and pulmonary arterial pressure) in patients with idiopathic and associated PAH and in animals with experimental PH 2, 3. The PI3K/AKT/mTOR signaling pathway, as one of the key signaling cascades to stimulate cell proliferation and survival, plays an important pathogenic role in the development and progression of PH 20, 44. Many G protein-coupled receptors (GPCRs), such as endothelin receptor A and Ca^2+^-sensing receptors, and tyrosine kinase receptors (TKRs), such as PDGFRs, are highly expressed in PA and PASMCs from patients with PAH 35, 45, 46. Activation of the up-regulated GPCR (e.g., endothelin receptor A and Ca^2+^-sensing receptors) and TKR (e.g., PDGFRα and PDGFRβ) by mitogenic factors and cytokines is one of the important mechanisms leading to abnormal cell growth, proliferation, and migration. Increased extracellular ligands (e.g., endothelin-1, spermine, PDGF) and up-regulated expression of various GPCRs (e.g., endothelin receptor A, Ca^2+^-sensing receptors) and TKRs (PDGFR) have been implicated in the development and progression of pulmonary vascular remodeling in patients with PAH 35, 46, 47. Pharmacological blockade or small interfering ribonucleic acid–mediated down-regulation of various GPCRs and TKRs in PASMCs, PAECs, and fibroblasts inhibits mitogen-mediated cell proliferation and attenuates the development and progression of PAH in patients and experimental PH in animals 48, 49, 50.

The present study found the following: 1) SM-specific KO of *mTOR* (*mTOR*^SM−/−^), which disrupts the function of both mTORC1 and mTORC2, significantly attenuates the development of hypoxia-induced PH (resulting in a 66% inhibition of hypoxia-induced increase in RVSP), but has a negligible effect on RVSP and the Fulton index under normoxic control conditions; 2) SM-specific KO of *Raptor* (*Raptor*^SM−/−^), which disrupts the function of mTORC1, also significantly attenuates the development of hypoxia-induced PH (resulting in a 28% inhibition of hypoxia-induced increases in RVSP) but has a negligible effect on RVSP and the Fulton index under normoxic control conditions; 3) SM-specific KO of *Rictor* (*Rictor*^SM−/−^), which disrupts the function of mTORC2, attenuates the development of hypoxia-induced PH (resulting in a 39% inhibition of hypoxia-induced increases in RVSP) but has little effect on hypoxia-induced PA wall thickening; 4) *Rictor*^SM−/−^ mice spontaneously develop mild PH, and RVSP and the Fulton index were increased, respectively, from 22.1 ± 0.14 mm Hg and 0.22 ± 0.02 to 30.5 ± 1.6 mm Hg (38.0% increase; p < 0.01) and 0.31 ± 0.06 (40.9% increase; p < 0.01) 3 months after tamoxifen injection and to 31.3 ± 1.3 mm Hg (41.2% increase; p < 0.001) and 0.31 ± 0.04 (40.9% increase; p < 0.001) 6 months after tamoxifen injection; and 5) the spontaneous increase in RVSP and the Fulton index in *Rictor*^SM−/−^ mice is associated with an up-regulation of PDGFRα and PDGFRβ in PA, and intraperitoneal injection of the PDGFR inhibitor imatinib reversed the established PH in *Rictor*^SM−/−^ mice.

These data indicate that the PI3K/AKT/mTOR signaling pathway plays an important pathogenic role in the development of PH. SM-specific KO of *Rictor* or dysfunction of mTORC2 has a paradoxical effect on the pulmonary vasculature, which attenuates experimental PH (or hypoxia-induced PH) via its inhibitory effect on AKT/mTORC1 signaling (mTORC2 is upstream of mTORC1 and positively regulates mTORC1 activity) and results in up-regulation of PDGFRα and PDGFRβ via phosphorylating AKT at S473 that contributes to a basal increase in RVSP and RVH.

Our previous studies indicated that phosphorylation of AKT in lung tissues and PASMCs was increased in patients with idiopathic PAH and in animals with experimental PH compared with controls; up-regulation of PTEN in *PTEN* transgenic mice attenuated experimental PH (implying a critical role for PI3K in the development of PH); global KO of the *Akt1* gene (*Akt1*^−/−^) inhibited the development of experimental PH and RVH, whereas global KO of the *Akt2* gene (*Akt2*^−/−^) had a negligible protective effect on hypoxia-induced PH and RVH (16); and genetic deletion of the *mTOR* gene (*mTOR*^−/−^) exerted a significant protective effect on hypoxia-induced PH and RVH in HPH mice. These observations implied that the PI3K/AKT1/mTOR signaling in PASMCs is required or significantly involved in the development of experimental PH.

The structural and functional complexities of mTORC1 and mTORC2, as well as their multiple protein interacting surfaces and regulatory role in PASMC proliferation and survival, make it difficult to rationalize which protein subunit should be considered to study in detail to define the potential differential effect of mTORC1 and mTORC2 on the development of PH 17, 51, 52. We have focused our experiments on the conserved proteins that have equivalent function in both complexes (mTORC1 and mTORC2) and are conserved in patients with PAH and in rodent PH models. Thus, we chose to investigate the pathogenic role of Raptor, a major protein subunit in the mTORC1 complex, and Rictor, a major protein subunit in the mTORC2 complex (53), in this study. There are multiple agonists that are identified to activate mTORC1 and mTORC2 by activating GPCR/TKR and eventually lead to cell proliferation and survival (54). Although the list of interacting proteins associated with mTORC1 and mTORC2 is growing, it is important to understand the individual upstream and downstream regulators of mTORC1 and mTORC2 in their potential pathogenic roles in the development of PH.

Both mTORC1 and mTORC2 are activated to induce angiogenesis and cell proliferation in response to hypoxia 44, 55, 56. Furthermore, studies have shown that mTORC1 has an early activating effect and late inhibitory effect on angiogenesis and cell proliferation in response to hypoxia, whereas mTORC2 only has a delayed and maintained activating effect on cell proliferation in response to hypoxia (44). In addition to cell proliferation and growth, mTORC1 is also involved in regulating protein synthesis, ribosome biogenesis, transcriptional control, and autophagy (57), whereas mTORC2 regulates the organization of the actin cytoskeleton and determines the motility and shape of the cell through Rho-type GTPases and protein kinase C (33). The upstream regulators of the mTORC1 pathway include various intracellular signals that are activated by growth factors, stress, energy metabolism, hypoxia and hyperoxia, and amino acids (58). The heterodimer TSC2 is a key upstream regulator of mTORC1 and functions as a GTPase-activating protein for Rheb (Ras homolog enriched in brain) (59). Loss of TSC2 increases cell proliferation and survival, and this effect also requires mTORC2 and its downstream effector Rho GTPase (20). Rapamycin inhibits the interaction of Raptor with other subunits in the mTORC1 complex, whereas Rictor, conversely, forms a rapamycin-insensitive complex with other components in mTORC2 (29). These findings suggest cooperative mechanisms between the signals from mTORC1 and mTORC2, and it becomes essential to understand the differential role played by mTORC1 and mTORC2 in stimulating cell proliferation and survival.

Many studies indicate that mTORC1 and mTORC2 function differently in regulating gene expression, cell proliferation, and growth (44). KO of *mTOR* would disrupt the function of both mTORC1 and mTORC2 (60). The goal of the present study was to examine the potential divergent or differential role of mTORC1 and mTORC2 in the development of PH. To achieve this goal, the protective effects of the following were compared: 1) the SM-specific and tamoxifen-inducible *Raptor* KO on pulmonary hemodynamic variables in *Raptor*^SM−/−^ mice in which the mTORC1 function is disrupted in PASMCs; and 2) the SM-specific and tamoxifen-inducible *Rictor* KO on RVSP and the Fulton index in *Rictor*^SM−/−^ mice in which the mTORC2 function is disrupted in PASMCs.

In consistent with our previously published data using *mTOR*^−/−^ mice (16), we found that SM-specific and tamoxifen-inducible KO of *mTOR* resulted in a 66% inhibition of HPH or hypoxia-induced increase in RVSP in *mTOR*^SM−/−^ mice. SM-specific KO of *Raptor* or induced inhibition of mTORC1 in PASMC (*Raptor*^SM−/−^) caused a 28% inhibition of hypoxia-induced increase in RVSP or HPH. Neither *mTOR*^SM−/−^ mice nor *Raptor*^SM−/−^ mice exhibited spontaneous PH or RVH under normoxic conditions. SM-specific KO of *Rictor* or induced inhibition of mTORC2 in PASMC (*Rictor*^SM−/−^) resulted in a 39% inhibition of HPH or hypoxia-induced RVSP increase in RVSP, but caused spontaneous PH or a 38% to 42% increase in RVSP under normoxic control conditions. The increase of the basal RVSP in *Rictor*^SM−/−^ mice was associated with an increased protein expression of PDGFRs (PDGFRα and PDGFRβ) in PA and PASMCs, and inhibition of PDGFR with imatinib reversed the established PH in *Rictor*^SM−/−^ mice.

The protein expression level of both PDGFRα and PDGFRβ in PASMCs isolated from patients with idiopathic PAH were greater than in PASMCs isolated from normal subjects. Furthermore, chronic hypoxia also up-regulated PDGFRα and PDGFRβ in PA and PASMCs isolated from animals. These data indicate that up-regulation of PDGFRs in PASMCs is also involved in the development of PAH/PH, due potentially to enhanced PASMC proliferation via a PDGF/PDGFR/PI3K/AKT/mTOR signaling pathway. The pathogenic effect of SM-specific *Rictor*-KO due to PDGFR up-regulation and the protective effect of SM-specific *Rictor*-KO due to mTORC2/AKT inhibition on experimental PH indicate that Rictor or mTORC2 has a paradoxical effect on the pulmonary vascular structure and function. The paradoxical effect of SM-specific KO of *Rictor* or inhibition of mTORC2 is also an important finding implying that combination use of inhibitors of mTORC2/AKT (and AKT/mTORC1) and inhibitors of PDGFRs would have a more efficient therapeutic or protective effect on PAH/PH.

## Conclusions

The PI3K/AKT1/mTORC1 signaling pathway is an important signaling cascade associated with GPCR/TKR–mediated PASMC proliferation and growth. The receptor-mediated AKT1 phosphorylation and the increase in mTORC1 kinase activity are critical in transferring the extracellular proliferative or mitogenic signals to the nucleus of PASMCs. Activation of the PI3K/AKT1/mTORC1 signaling cascade plays an important pathogenic role in the development of pulmonary vascular remodeling and PH. Down-regulation or inhibition of mTOR (required for the function of mTORC1 and mTORC2), Raptor (required for the function of mTORC1), and Rictor (required for the function of mTORC2) ameliorate the experimental PH in mice. In addition to inhibiting hypoxia-induced PH, however, down-regulation of *Rictor* or inhibition of mTORC2 also up-regulates PDGF receptors in PASMCs, whereas *Rictor*^SM−/−^ mice exhibit spontaneous PH that can be reversed by inhibition of PDGFRs (with imatinib). Because the inhibition of mTORC2 results in a paradoxical effect of experimental PH, we suggest that therapeutic regimens using inhibitors of the PI3K/AKT/mTOR signaling cascade for the treatment of PH and PAH should include an inhibitor of PDGFR (e.g., imatinib) due to the up-regulation of PDGFRα and PDGFRβ induced by mTORC2 inhibition.

Perspectives **COMPETENCY IN MEDICAL KNOWLEDGE:** Idiopathic PAH is a fatal and progressive disease characterized by increased PVR creating strain on the right ventricle that can progress to right heart failure and death. Current therapies remain insufficient with the absence of effective disease-modifying or preventive interventions, and application of most of the current agents is hampered by undesirable side effects. Our study is the first to report a promising therapeutic strategy of combination treatment with an inhibitor of PDGFR and an inhibitor of mTOR on PAH. We show that inhibition of mTORC1 ameliorates experimental PH, whereas inhibition of mTORC2 up-regulates PDGFR in PASMCs and compromises the therapeutic effect of mTOR inhibition on PH. Furthermore, we show that intraperitoneal injection of imatinib completely reverses the established PH in *Rictor*-KO mice, whereas the combination therapy with rapamycin and a low dose of imatinib dramatically reverses the established severe PH in rats (hypoxia/Sugen–induced PH); rapamycin alone only caused 25.7% inhibition.

**TRANSLATIONAL OUTLOOK:** The data from these in vitro and in vivo animal studies, although with limitations, strongly suggest a combination therapeutic strategy using inhibitors of mTOR and PDGFRs for PAH and PH associated with hypoxia and lung diseases.
