# Supplementary material for: Pathogenic Role of mTORC1 and mTORC2 in Pulmonary Hypertension
Source: JACC Basic Transl Sci. 2018 Dec 31;3(6):744–62. doi: 10.1016/j.jacbts.2018.08.009 (PMC6314964; doi:10.1016/j.jacbts.2018.08.009)
Supplement: Supplemental Material [file mmc1.pdf]

ONLINE APPENDIX

Methods and Materials

Animals. Wild-type (WT) control mice and knockout (KO) mice with C57BL/6 background were used for the in vivo animal experiments. Mice at age of 6-12 weeks old were used for the pharmacological and therapeutic experiments. We used Cre-Lox technology to generate a smooth muscle (SM)-specific inducible KO mouse line. *Raptor* floxed mice, herein called *Raptor*$^{\text{flox/flox}}$ (*Raptor*$^{\text{F/F}}$) were crossed with *SMMHC-CreER*^{T2} mice for two generations to create *SMMHC-CreER*$^{\text{T2}}$/*Raptor*$^{\text{flox/flox}}$ (*SMMHC-CreER*$^{\text{T2}}$/*Raptor*$^{\text{F/F}}$) mice. In this mouse strain, a tamoxifen-inducible Cre-recombinase is under the control of the smooth muscle myosin heavy chain (SMMHC) promoter. Similar technique was used to create *SMMHC-CreER*$^{\text{T2}}$*/Rictor*$^{\text{flox/flox}}$ (*SMMHC-CreER*$^{\text{T2}}$/*Rictor*$^{\text{F/F}}$) mice. The reason for employing an inducible recombination strategy was that it allowed for the determination of the consequences of functional loss of *Raptor* (mTORC1) or *Rictor* (mTORC2) in the adult mouse PASMC. Mice were treated with tamoxifen (cat. No. T5648; Sigma Aldrich) via intraperitoneal injection (i.p.) once a day for five consecutive days at a dose of 1 mg to induce gene KO.

Genotyping. DNA was extracted from tail biopsy samples following standard procedures to genotype various mouse strains. Genotyping of homozygous and heterozygous traits were confirmed by PCR. The primer sequences and anticipated band sizes are as follows: mTOR (forward): 5’-TTA TGT TTG ATA ATT GCA GTT TTG GCT, mTOR (reverse): 5’-TTT AGC ACT CCT TCT GTG ACA TAC ATT; Raptor (forward): 5’-CTC AGT GGT ATG TGC TCA G-3’, Raptor (reverse): 5’-GGG TAC AGT ATG TCA GCA CAG-3’; Rictor (forward): 5’-CAA GCA TCA TGC AGC TCT TC-3’, Rictor (reverse): 5’-TCC CAG AAT TTC CAG GCT TA-3’.

The length of the PCR products of WT and mutant (Mut) for the mice are as follows: mTOR-WT, 349 bp; mTOR-Mut, 533 bp; mTORC1 (Raptor)-WT, 141 bp; mTORC1 (Raptor)-Mut, 180 bp; mTORC2 (Rictor)-WT, 389 bp; mTORC2 (Rictor)-Mut, 500 bp. The cycling parameters for the PCR were as follows: 1 cycle at 94°C for 5 min, 33 cycles of 94°C for 45 sec, then 45 sec of 62°C, and 72°C for 45 sec. The PCR ended with 5 min at 72°C.

Pulmonary Hypertension Models and Hemodynamic Measurements. In the hypoxic pulmonary hypertensive model, rats and mice were placed in normobaric hypoxic chambers (10% O_2, BioSperix A chambers; BioSperix, Lacona, NY)) for 3-5 weeks as previously described (1). The normoxic control animals were exposed to room air (21% O_2) for the same period of time. Periodically, partial pressure of oxygen (PO_2) was monitored with a Proox Model P110 oxygen controller (BioSperix). At the end of 3-5 weeks of chronic exposure to normobaric hypoxia, the rodents were anaesthetized with ketamine/xylazine before measurement of hemodynamics. The procedure for rat models of PH induced by hypoxia and Sugen (Hyp+SU) is similar to the model of hypoxia-induced PH (HPH), but with the addition of subcutaneous injection of SU5416, a vascular endothelial growth factor (VEGF) receptor or protein tyrosine kinase 1/2 inhibitor that has been shown to cause sever PH (2). One dose of SU5416 (20 mg/kg body weight; Sigma-Aldrich Corporation, Milwaukee, WI) was given at the first day of hypoxic exposure. After 3 weeks of hypoxic exposure, animals were moved from the hypoxic chambers and relocated to normoxic conditions for additional 2 weeks. For pharmacological experiments, the treated groups included rats that received once-daily i.p. injection of imatinib (LC Laboratories, Woburn, MA) at a dose of 20 mg/kg, rapamycin (ThermoFisher, Pittsburgh, PA) at a dose of 5 mg/kg, two agents in combination (imatinib, 20 mg/kg, and rapamycin, 5 mg/kg), or the administration vehicle

(isotonic saline) after hypoxic exposure. Rats were examined after 14 days of treatment under normoxic conditions. The weight of the animals was measured before the experiment and once a week during the experiments. Right ventricular pressure (RVP) and right ventricle (RV) contractility (RV-$\pm dP/dt_{max}$) were measured by a 1.4F transducer catheter (Millar Instruments. Houston, TX), recorded and analyzed by the AcqKnowledge software (Biopac Systems, Inc., Goleta, CA). Right ventricular systolic pressure (RVSP) was used to estimate the pulmonary arterial systolic pressure. Fulton Index, the ratio of the weight of RV to the weight of left ventricle (LV) and septum (S) [RV/(LV+S)], was measured when the whole-heart was excised and dissected after hemodynamic measurement.

Isolation of Mouse Pulmonary Artery. The intrapulmonary arteries were carefully dissected out from mouse lungs as described previously (3). Briefly, the whole lung and heart were removed from the mouse and placed in Hank's balanced salt solution (HBSS, Life Technologies, Carlsbad, CA). The right and left branches of the intrapulmonary arteries were isolated from the whole-lung with fine forceps under a dissecting microscope. The connective and fat tissues were gently removed from the isolated pulmonary artery. Endothelium was functionally denuded by inserting a human hair to the isolated pulmonary artery to scratch the intraluminal surface for 3-5 times. The isolated pulmonary artery was then used to extract total protein for Western blot experiments.

Immunohistochemistry. Immunohistochemical staining was performed for detecting the mTOR, Raptor and Rictor proteins in the lung tissue sections. Fixed lung tissue blocks were embedded in paraffin and cut to 2-µm thickness slice and mounted on the tissue slides. Tissue cross-section slides were deparaffinized using Xylene and ethanol following the protocol [100% Xylene: 2×3

min; Xylene: ethanol (1:1): 3 min; 100% ethanol: 2×3 min; 95% ethanol: 3 min; 70% ethanol: 3 min; 50% ethanol: 3 min and finally the slides were kept in running tap water to avoid drying]. Heat mediated antigen retrieval was done with Sodium Citrate Buffer (10 mM sodium citrate, 0.05% Tween 20, pH 6.0). Tissues were blocked using 5% BSA in PBS for 1 hr in a dark humidified chamber. Tissues were washed with PBST (Phosphate buffered saline and Tween 20) 3 times for 10 min each, and incubated with primary antibody overnight in the cold room. After overnight incubation, tissues were washed and incubated for 2 hrs at room temperature with secondary antibody which is fluorophore tagged. Tissues were then washed again and incubated with smooth muscle α-actin (SMA) antibody for 2 hrs at room temperature. The tissues were finally washed 3 times with PBST and mounted with mounting medium which contains 6'-diamidino-2-phenylinodole (DAPI) (Prolong ® Gold Antifade Reagent with DAPI, Carlsbad, CA). The FITC (Fluorescein Isothiocyanate) fluorescence was colored green, Cy3 fluorescence was colored red and DAPI fluorescence was colored blue to display images with green, red and blue overlay. Green fluorescence emitted at 509 nm was used to detect mTOR, mTORC1 (Raptor) and mTORC2 (Rictor) protein complex. Red fluorescence emitted at 562 nm was used to detect SMA. Blue fluorescence emitted at 463 nm was to detect the nuclei. Primary antibodies included the antibodies against mTOR (both mTORC1 and mTORC2) (1:200, Thermo Scientific, MA), Raptor (mTORC1) (1:200, Abcam, Cambridge, MA), and Rictor (mTORC2) (1:200, Cell Signalling, Danvers, MA). Secondary antibody used in the experiments was fluorophore-tagged anti-rabbit antibody (1:200). Fluorescence was captured by Zeiss confocal microscope with 20× objective lens. Exposure time was set constant across the images. The fluorescence expression was quantified using Image J analysis, normalized to control between two groups (Oil and Tamoxifen induced) and the P value was calculated by standard statistical test to look for any significance.

Culture of PASMC. Human PASMC from normal subjects (Lonza, Walkersville, MA and PHBI，Philadelphia, PA) and IPAH patients (PHBI, Philadelphia, PA) were cultured in smooth muscle growth media (SMGM, Lonza, Walkersville, MA) supplemented with 5% fetal bovine serum (FBS), 0.5 ng/ml human epidermal growth factor (EGF), 2 ng/ml human fibroblast growth factor (FGF), and 5 μg/ml insulin. Human PAEC were cultured in endothelial growth medium (EGF, Lonza, Walkersville, MA) supplemented with 2% FBS, human EGF, human FGF and insulin. The cells at passage 5-8 were used for the experiments. The human PASMC and PAEC were cultured in an incubator under a humidified atmosphere of 5% CO_2 and 95% air at 37°C.

RT-PCR. Total RNA was isolated from PASMC or PAEC with TRIzol reagent. 1 mg of total RNA was used for reverse transcription with Taq Man Reverse Transcription Reagents (Applied Biosystems), which was quantitated by NanoDrop (Thermo Scientific). The emerging first-strand cDNA was used as a template for PCR reaction with High Fidelity Master Mix (New England Biolabs). PCR primers were designed using the NCBI Primer Blast and synthesized by Integrated DNA Technologies. The primer sequences and anticipated band sizes are as follows: PDGFRα (forward): 5’- AGG GAT AGC TTC CTG AGC CA, PDGFRα (reverse): 5’- GAC ATC TCG TGC CAA CTC CA. PDGFRβ (forward): 5’- GTG AGC AAC TTG GAG CCA GA, PDGFRβ (reverse): 5’- GTC CCA GAG TGG GTA ACA GC . RT-PCR results were resolved using 2% agarose gels and ethidium bromide staining.

Western blot analysis. Lung tissues or whole cells protein isolated using Ripa Lysis Buffer (Pierce RIPA Product, Rockford,IL) supplemented with protease inhibitor cocktail (Roche; Manheim,

Germany) by sonification 3 times for 30 sec each time. Nuclear and cytoplasmic protein fractionation was performed using NE-PER Nuclear and Cytoplasmic extraction kit (Thermo Fisher Scientific, Rockford, IL). Protein concentration was determined by Bradford Protein Assay (Bio-Rad; Hercules, CA) with BSA as a standard. Protein samples were mixed and boiled with Ripa Lysis Buffer supplemented with 6× Dye (Bio-Rad Laemmli Sample Buffer, CA). Protein lysates were resolved on sodium dodecyl sulphate polyacrylamide gel electrophoresis (SDS-PAGE) and transferred onto 0.45 μm nitrocellulose membranes (Bio-Rad). Membranes were incubated for 1hr at room temperature in a blocking buffer (0.1% Tween 20 in TBS (TBST)) containing 5% nonfat dry milk powder. The membranes were then incubated with primary antibodies diluted in TBST containing 5% BSA, shaking overnight at 4°C. Membranes were washed 3 times in TBST for 5 min each, followed by incubation in secondary antibody for 2 hrs at room temperature in TBST containing 5% milk. Membranes were washed 4 times for 5 min each and peroxidase activity was visualized with enhanced chemiluminescence substrate. (Pierce; Rockford, IL). Primary antibodies used in the experiments included rabbit Raptor antibody (Cell Signalling, Danvers, MA, 1:1000), rabbit Rictor antibody (Cell Signalling, Danvers, MA, 1:1000), rabbit AKT antibody (Cell Signalling, Danvers, MA, 1:1000), rabbit p-AKT(S473) antibody (Cell Signalling, Danvers, MA, 1:1000), rabbit p-AKT (T308) antibody (Cell Signalling, Danvers, MA, 1:1000), rabbit PDGFRα antibody(Santa Cruz Biotechnology, CA, USA, 1:1000), rabbit PDGFRβ antibody(Santa Cruz Biotechnology, CA, USA, 1:1000) and mouse β-actin antibody (Santa Cruz Biotechnology, CA, USA, 1:5000). Band intensity was quantified with Image J, normalized to β-actin control.

Statistics. Summarized data are expressed as means±standard error (SE). Statistical significant differences were assessed between groups using standardized statistical analyses. Differences were considered to be significant at $p<0.05$. Statistical analyses were performed using SigmaPlot 11.0 (Systat Software, San Jose, California) and R (R-project, version3.32). Statistical differences were assessed with unpaired Student's *t*-test except when there was heterogeneity of variance and then a Welch's *t*-test was run using R (4). Otherwise, statistical significance was determined using the Kruskal-Wallis test followed by a Dunn test for post-hoc pairwise comparison when significant. All *p* values <0.05 were considered statistically significant.

References

1. Tang H, Babicheva A, McDermott KM et al. Endothelial HIF-2α contributes to severe pulmonary hypertension due to endothelial-to-mesenchymal transition. *Am J Physiol Lung Cell Mol Physiol* 2018; 314-L256-L275.
2. Taraseviciene-Stewart L, Kasahara Y, Alger L et al. Inhibition of the VEGF receptor 2 combined with chronic hypoxia causes cell death-dependent pulmonary endothelial cell proliferation and severe pulmonary hypertension. *FASEB J* 2001;15:427-38.
3. Wang J, Shimoda LA, Weigand L, Wang WQ, Sun DJ, Sylvester JT. Acute hypoxia increases intracellular [Ca^{2+}] in pulmonary arterial smooth muscle by enhancing capacitative Ca^{2+} entry. *Am J Physiol Lung Cell Mol Physiol*. 2005;288:L1059-L1069.
4. Welch BL. The generalization of "Student's" problem when several different population variances are involved. *Biometrika* 1947; 34:28-35.
